# An orthogonal dual-regulation strategy for sensitive biosensing applications

**DOI:** 10.1093/nsr/nwac048

**Published:** 2022-03-12

**Authors:** Xian Yang, Jinhua Wang, Zhongfeng Gao, Weiqi Zhang, Hai Zhu, Yongjun Song, Quan Wang, Mingjie Liu, Lei Jiang, Yu Huang, Fan Xia

**Affiliations:** State Key Laboratory of Biogeology and Environmental Geology, Engineering Research Center of Nano-Geomaterials of Ministry of Education, Faculty of Material Science and Chemistry, China University of Geosciences, Wuhan 430074, China; State Grid Integrated Energy Service Group CO. LTD., Beijing 100052, China; State Key Laboratory of Biogeology and Environmental Geology, Engineering Research Center of Nano-Geomaterials of Ministry of Education, Faculty of Material Science and Chemistry, China University of Geosciences, Wuhan 430074, China; State Key Laboratory of Proteomics, Beijing Proteome Research Center, National Center for Protein Sciences (Beijing), Beijing Institute of Lifeomics, Beijing 102206, China; State Key Laboratory of Biogeology and Environmental Geology, Engineering Research Center of Nano-Geomaterials of Ministry of Education, Faculty of Material Science and Chemistry, China University of Geosciences, Wuhan 430074, China; Shandong Provincial Key Laboratory of Detection Technology for Tumor Markers, College of Chemistry and Chemical Engineering, Linyi University, Linyi 276005, China; State Key Laboratory of Biogeology and Environmental Geology, Engineering Research Center of Nano-Geomaterials of Ministry of Education, Faculty of Material Science and Chemistry, China University of Geosciences, Wuhan 430074, China; State Key Laboratory of Biogeology and Environmental Geology, Engineering Research Center of Nano-Geomaterials of Ministry of Education, Faculty of Material Science and Chemistry, China University of Geosciences, Wuhan 430074, China; State Key Laboratory of Biogeology and Environmental Geology, Engineering Research Center of Nano-Geomaterials of Ministry of Education, Faculty of Material Science and Chemistry, China University of Geosciences, Wuhan 430074, China; State Key Laboratory of Biogeology and Environmental Geology, Engineering Research Center of Nano-Geomaterials of Ministry of Education, Faculty of Material Science and Chemistry, China University of Geosciences, Wuhan 430074, China; Key Laboratory of Bio-Inspired Smart Interfacial Science and Technology of the Ministry of Education, School of Chemistry and Environment, Beihang University, Beijing 100191, China; Key Laboratory of Bio-Inspired Smart Interfacial Science and Technology of the Ministry of Education, School of Chemistry and Environment, Beihang University, Beijing 100191, China; State Key Laboratory of Biogeology and Environmental Geology, Engineering Research Center of Nano-Geomaterials of Ministry of Education, Faculty of Material Science and Chemistry, China University of Geosciences, Wuhan 430074, China; Zhejiang Institute, China University of Geosciences, Hangzhou 311305, China; State Key Laboratory of Biogeology and Environmental Geology, Engineering Research Center of Nano-Geomaterials of Ministry of Education, Faculty of Material Science and Chemistry, China University of Geosciences, Wuhan 430074, China; Zhejiang Institute, China University of Geosciences, Hangzhou 311305, China

**Keywords:** motion behavior, sensitive detection, dynamic range, hydrophobic interaction, anisotropic resistance

## Abstract

Biosensing systems based on controllable motion behaviors of droplets have attracted extensive attention, but still face challenges of insufficient sensitivity and uncontrollable dynamic range due to imprecise manipulation of droplet motion on the surfaces. Here, we report an orthogonal dual-regulation strategy for precise motion control of droplets and we demonstrate its utility as a sensitive sensing system with controllable dynamic ranges of sensing for adenosine triphosphate, miRNA, thrombin and kanamycin, as well as discrimination of five kinds of DNA. We endowed a DNA-contained bio-droplet sliding on a lubricant-infused structural surface with micro-grooves to separately adjust the resistance from liquid phase and solid phase. The resistance from liquid phase mainly depended on hydrophobic interaction between DNA and lubricant, which can be finely tuned by different DNA’s average chain length. Meanwhile, the resistance from solid surface was determined by the energy barrier from the periodic micro-grooves, which can be adjusted by varying the droplet's sliding direction on the surface. The hydrophobic interaction is conformed to be orthogonal to the micro-grooves’ anisotropic resistance by three different methods. This orthogonal dual-regulation strategy thus demonstrated its ability to precisely control bio-droplets’ motion behaviors and sensitive detection with adjustable dynamic ranges for various bio-targets. The dual-regulation strategy will provide significant insights for super-wettable biosensors, visual inspection and beyond.

## Teaser

An orthogonal dual-regulation strategy was developed for droplets’ precise motion control, and applied as a sensitive sensing system with controllable sensing dynamic ranges for various bio-markers.

## INTRODUCTION

Precise control of a droplet's motion behaviors on solid surfaces is fundamental in unidirectional water transportation, microfluidic devices, liquid harvesting, transportation, drag reduction and numerous related processes [1–7]. Research on the wettability and adhesion mechanisms of substrates has promoted the development of droplets' motion control on various surfaces and relative detection systems. Among them, lubricant-infused surfaces with outstanding advantages of self-cleaning, antifouling, optical focusing, power generation and biosensing applications have been well developed and increasingly attracted research interest [8–15]. During the past decade, we also reported a class of sensing systems with the concept of controlling the droplets’ motion behavior by adjusting the resistance from the solid phase, mainly through constructing wettability gradients and adopting responsive surfaces [16–22]. Gradient wettability surfaces display physicochemical characteristics changing in one dimension and are capable of transporting water droplets efficiently. For responsive surfaces, external stimuli, including mechanical stretching [23], thermos [24,25], electric field [26], magnetic field [27], light [28,29], pH [30] and so on [31,32] are required. Therefore, controlling of a droplet's motion behavior can be realized by developing new surface fabrication technologies to adjust resistance from the solid phase.

Recently, we demonstrated that the bio-droplets' motion behavior also can be controlled by manipulation of the resistance from the liquid phase [33]. Through adjusting hydrophobic interaction between biological droplets and lubricants by DNA’s average chain length, controllable critical sliding angle (CSA) of the bio-droplet was realized. As a result, detecting targets with 10-fold change in their concentrations can be achieved, with its non-adjustable dynamic range of 10^5^-fold. In our experiment, the dynamic range is defined as the range of a target's concentration with relative CSA occupancy of between 10% and 90% (more information on this can be found in Supporting Information) [34]. Despite significant efforts having been achieved in a controllable droplet's motion behavior, separate manipulation of the resistance from solid and liquid is rare via a single strategy. Therefore, the wettability-based detection systems still face insufficient sensitivity and uncontrollable dynamic range. Based on the manipulation of liquid-phase resistance, designing another tunable resistance that is orthogonal to liquid-phase resistance for droplets’ precise motion control and sensitive detection is highly desired.

**Figure 1.**
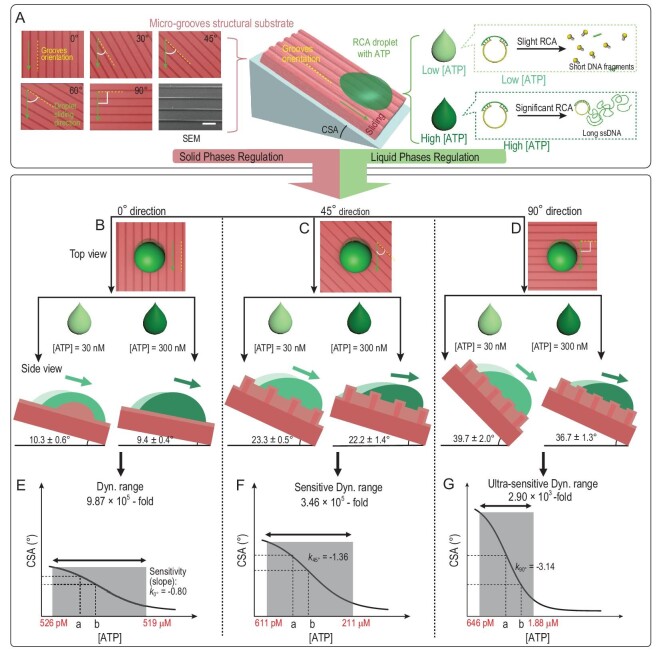
Illustration of the dual-regulation strategy for precise control of motion behaviors of droplets and its sensing applications. (A) Illustration of dual-regulation for controllable sliding behavior of RCA droplets (the scale bar is 250 μm). (B)–(D) RCA droplets with ATP concentrations of 30 and 300 nM sliding in 0°, 45° and 90° directions, respectively, displaying tunable detection sensitivity and adjustable dynamic ranges. (E)–(G) When the sliding direction changes from 0°, 45° to 90°, the dynamic range narrows down and the sensitivity (slope) increases, contributing to the distinguishing of droplets with a smaller change in the target's concentration. Numbers in red are the upper and lower limits of dynamic ranges. The ‘a’ and the ‘b’ represent concentrations of ATP of 30 and 300 nM, respectively.

Here, we address this issue by incorporating manipulation of liquid-phase resistance and orthogonal tunable solid resistance in a single strategy. Through separately manipulating the resistance from solid and liquid phases, ultra-sensitive detection based on precise control of biological droplets’ motion behavior was achieved (Fig. 1). For liquid-phase manipulation, by designing an applicable probe and primer (Table S1), adenosine triphosphate (ATP) can induce rolling-circle amplification (RCA) and then tune the hydrophobic interaction between the RCA droplet and lubricant. With a high concentration of ATP, significant RCA proceeded to increase the average chain length of DNA and decrease the relative hydrophobic interaction. Therefore, the RCA droplet was sliding easily on the tilted surface. For solid-phase manipulation, lubricant-infused polydimethylsiloxane (PDMS) substrates with a periodic micro-groove structure were selected. According to the literature, lubricant must stably adhere within the substrate and be immiscible with the test droplet [8]. In our experiment, n-decane was adopted as the lubricant. When the RCA droplet was sliding on the substrates in different directions, the resistance acted on the droplet from the solid phase can be adjusted. In this work, the resistance from the substrate results from an energy barrier caused by the micro-grooves. The hydrophobic interaction was confirmed as orthogonal to the micro-grooves’ anisotropic resistance by three different methods. As a result, when RCA droplets with various ATP concentrations are sliding in different directions on the surface, their motion behaviors can be well controlled and analysed. In this manuscript, as shown in the left part of Fig. 1A, the sliding direction was defined as the included angle between the droplet's sliding direction (green arrow) and the orientation of the micro-grooves (yellow dashed line) on the substrate. Meanwhile, various relationships between CSAs and ATP concentrations with sensitivity that changed from –0.80 to –3.14 were also realized, demonstrating an adjustable sensitivity of detection (Fig. 1B–G). Here, sensitivity was defined as the change in response per unit change in target concentration and was represented as the slope of the linear fitting line of the dynamic range (for more information, please see Supporting Information).

**Figure 2.**
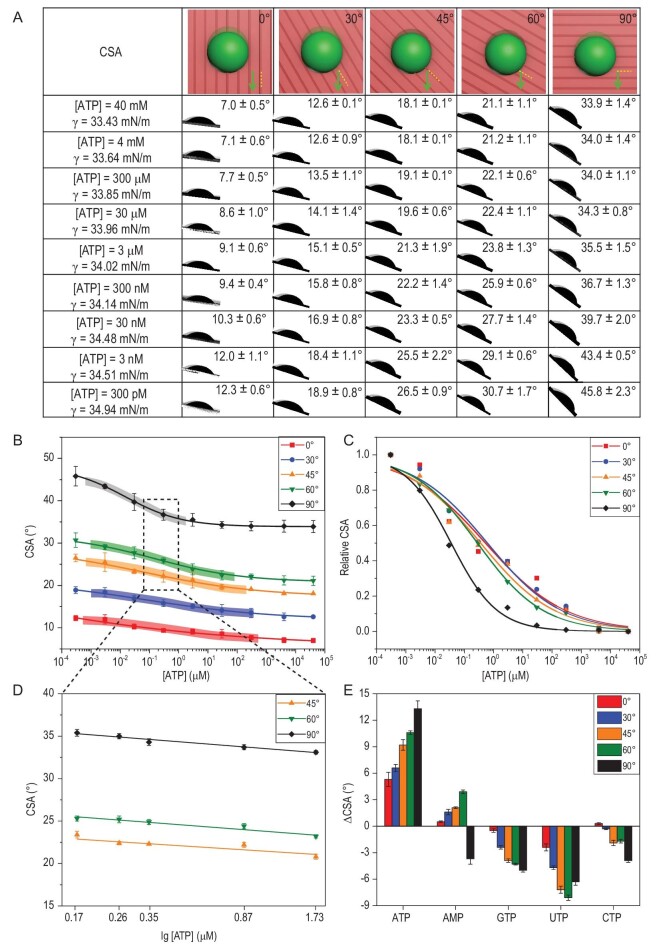
Precise controllable droplet-motion behaviors based on the dual-regulation strategy. (A) and (B) The relationships between CSAs and ATP concentrations (300 pM, 3 nM, 30 nM, 300 nM, 3 μM, 30 μM, 300 μM, 4 mM and 40 mM) of RCA droplets on periodic micro-groove slippery surfaces with sliding directions of 0°, 30°, 45°, 60° and 90°, respectively. The colorful shadows are the dynamic ranges of detection ATP in different sliding directions. (C) Normalized binding curves for different ATP concentrations and CSAs fitted by the Hill equation. (D) CSA response to the various ATP concentrations (0.17, 0.26, 0.34, 0.87 and 1.73 μM) in sliding directions of 45°, 60° and 90°, respectively. Distinguishing RCA droplets with a 2-fold change in ATP concentration of ∼300 nM was realized. (E) Changes in CSA with ATP and its analogs in five different sliding directions, displaying the great detection specificity of ATP.

Specifically, when the RCA droplets were sliding in a 90° direction (Fig. 1G), they met the greatest resistance from the micro-grooves and displayed obvious contact angle (CA) hysteresis. In this case, the most sensitive detection (the steepest curve from relationship between the ATP concentration and the CSA with slope of –3.14) with the narrowest dynamic range of 2.90 × 10^3^-fold was achieved. This ultra-sensitive detection displayed the ability of well-distinguished RCA droplets with a 2-fold ATP concentration change near 300 nM, i.e. 1.735 and 0.868 μM (Fig. 2D). Furthermore, other sensitive biosensing applications, including the detection of miRNA, thrombin and kanamycin, and the distinguishing of droplets with a 2-fold change in the target's concentration were also realized in their sensing dynamic ranges, respectively. Meanwhile, successful recognition of five DNAs was also demonstrated based on CSAs analysis. The dual-regulation strategy will be of great importance for designing and developing various advanced detecting systems with controllable dynamic range and sensitivity, which will provide significant insights for applications of super-wettable biosensors, visual inspection and beyond. The dual-regulation strategy is suitable for the wider scientific community. In addition, the output signal of the dual-regulation strategy can be easily recognized by the naked eye, providing a favorable way for users with color blindness or color weakness or in a situation with poor image display.

## RESULTS

### Dual-regulation from liquid and solid phases for precise control of RCA droplets’ motion behaviors

To demonstrate that the sliding behaviors of RCA droplets can be precisely controlled through this dual-regulation strategy, the RCA droplets triggered by different ATP concentrations were fabricated and their motion behaviors on slippery surfaces with periodic micro-grooves in different sliding directions were also tested. The substrates with a height of 20 μm, a width of 20 μm and a spacing of 200 μm were adopted in the experiments due to their stable and obvious anisotropic sliding property (Figs S1–S3). The pre-experimental results show that the concentration of the target in the liquid phase and the sliding direction on the substrate contribute greatly to the sliding behavior of the droplet (Fig. S4). In our experiments, the volumes of RCA droplets were all 2.0 μL. Meanwhile, there was a subtle change in the viscosity of as-prepared RCA droplets with different ATP concentrations (Fig. S5). Although the selection of lubricant and volume of RCA droplet would vary their motion behaviors, it would not affect the principal of orthogonal dual-regulation strategy (Figs S6 and S7). The flexible choice of lubricants and patterned substrates allows a large space for designing various sensing systems with tunable dynamic range and sensitivity.

### ATP detection with tunable sensitivity and adjustable dynamic ranges based on the RCA droplets’ motion behaviors on anisotropic slippery surfaces

Based on a dual-regulation strategy for controllable motion behaviors, CSAs of RCA droplets with various ATP concentrations and sliding directions were systematically characterized to demonstrate sensitivity detection with adjustable dynamic ranges, as shown in Fig. 1A. Five different sliding directions of the droplet were selected, including 0°, 30°, 45°, 60° and 90° to the orientation of the micro-grooves. When the concentration of ATP decreased from 40 mM to 300 pM, the CSAs increased in all five sliding directions: from 7.0 ± 0.5° to 12.3 ± 0.6° (second column in Fig. 2A), from 12.6 ± 0.1° to 18.9 ± 0.8°, from 18.1 ± 0.1° to 26.5 ± 0.9°, from 21.1 ± 1.1° to 30.7 ± 1.7° and from 33.9 ± 1.4° to 45.8 ± 2.3° for sliding directions of 0°, 30°, 45°, 60° and 90°, respectively (Fig. 2A). In the case of RCA droplets with the same ATP concentration but sliding in different directions, their CSAs increased from 0° to 90° due to the increased resistance from the micro-grooves. These results demonstrated that the dual-regulation strategy adjusted the resistance from solid phase and liquid phase separately. Finally, the CSAs of RCA droplets changed from 7.0 ± 0.5° (the first cell at the top in Fig. 2A) to 45.8 ± 2.3° (the last cell on the bottom), displaying precise control of droplet-motion behavior and adjustable sensitivity of ATP detection based on CSAs.

Relationships between CSAs and ATP concentrations of RCA droplets sliding in five different directions and their corresponding normalized CSAs curves are shown in Fig. 2B and C. With adjusted *R*-squares of >0.95, various dynamic ranges were achieved in different sliding directions. Meanwhile, the sensitivity of detection increased with sliding directions that changed from 0°, 30°, 45°, 60° to 90°, with corresponding slopes that gradually changed from –0.80, –1.01, –1.36, –1.76 to –3.14, respectively (Table S2).

In order to demonstrate the tunable sensitivity and adjustable dynamic range of detection in different sliding directions, a series of RCA droplets with ATP concentrations of ∼300 nM were selected, including 1.735, 0.868, 0.347, 0.260 and 0.174 μM. Their corresponding CSAs were almost the same in 0° and 30° sliding directions. In contrast, in sliding directions of 45°, 60° and 90°, the CSAs were changed for RCA droplets with a small change in ATP concentration, as shown in Fig. 2D, e.g. 9.0 ± 0.5°, 9.1 ± 0.6°, 8.9 ± 0.7°, 9.0 ± 0.5°, 8.9 ± 0.7° and 33.1 ± 0.2°, 33.7 ± 0.3°, 34.3 ± 0.4°, 35.0 ± 0.3°, 35.4 ± 0.4° for 0° and 90° sliding directions, respectively. The results were plotted as the normalized CSAs curves in the direction of 45° (3.46 × 10^5^-fold dynamic range), 60° (8.17 × 10^4^-fold dynamic range) and 90° (2.90 × 10^3^-fold dynamic range) steeper than that in the 0° (9.87 × 10^5^-fold dynamic range) and 30° (3.59 × 10^5^-fold dynamic range) directions, suggesting greater sensitivity of detection. Therefore, in the sliding directions of 45°, 60° and 90°, identification of RCA droplets with a 2-fold change in the ATP concentration of ∼300 nM was realized. Furthermore, to demonstrate the specificity of ATP towards the lubricant-infused slippery surface, other four ATP analogs, including adenosine monophosphate (AMP), guanosine-5^′^-triphosphate disodium salt (GTP), uridine triphosphate (UTP) and cytidine triphosphate (CTP), were also detected. As shown in Fig. 2E, compared with ATP, the droplet's mobility was displayed as barely changed or having an opposite change trend when the four analogs were added, indicating that the motion of the RCA droplet could be selectively manipulated by ATP. However, compared with the control group (without ATP), the amphiphilic analogs provided extra hydrophobic interaction. It is worth noting that in the 90° sliding direction, excellent specificity was realized due to the additional resistance from the structural substrate with micro-grooves.

**Figure 3.**
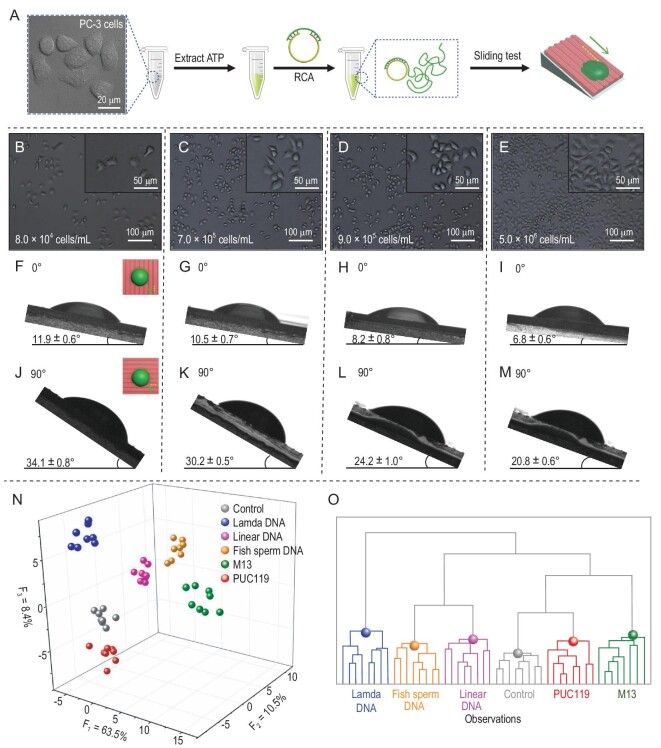
Droplet motion applied for a cell's reproduction-rate monitoring and discrimination of five DNAs. (A) Scheme of a cell's reproduction-rate monitoring based on analysing RCA droplets’ motion behaviors. (B)–(E) Bright-field images of PC-3 cells with different densities: (B) 8.0 × 10^4^, (C) 7.0 × 10^5^, (D) 9.0 × 10^5^ and (E) 5.0 × 10^6^ cells/mL. (F)–(M) Photos of RCA droplets triggered by corresponding cellular ATP sliding in (F)–(I) 0° sliding direction and (J)–(M) 90° sliding direction, respectively. (N) Recognition of five DNAs and one control sample based on CSAs and wetting-property analysis. The dispersion factors (*F*_1_–*F*_3_) are used to display the discriminative analysis and form the 3D representation of the various analyte clusters. (O) HCA image displaying the similarity clustering of the analytes.

The dual-regulation strategy and sensitive detection can be applied to monitor a cell's reproduction rate, which is directly proportional to the ATP level [35]. In our experiment, PC-3 cells with different densities of 8.0 × 10^4^, 7.0 × 10^5^, 9.0 × 10^5^ and 5.0 × 10^6^ cells/mL were achieved after incubation for 6, 36, 48 and 70 h. Then, intracellular ATP was extracted from each PC-3 live-cell lysate with a fixed volume of 1 mL. As shown in Fig. 3A–M, after the RCA droplets were triggered by corresponding intracellular ATP, CSAs were tested in 0° and 90° directions. When the density of the PC-3 cell increased from 8.0 × 10^4^ to 5.0 × 10^6^ cells/mL, the RCA droplets’ CSAs decreased from 11.9 ± 0.6° to 6.8 ± 0.6° in the 0° direction and significantly decreased from 34.1 ± 0.8° to 20.8 ± 0.6° in the 90° direction. The change in CSAs contributes to a sensitive monitoring of a cell's reproduction rate.

Meanwhile, the dual-regulation strategy also can be devoted to multiplex DNA discrimination. Obtaining enough differential and correlative signals is the key point for multi-targets analysis [36]. Five kinds of DNA with different chain lengths were selected to process the multi-target analysis, including Lambda DNA, Linear ssDNA, Fish Sperm DNA, PUC119 and M13, with Tris-EDTA buffer solution (TE buffer) as the control sample. CSAs, receding angles (*θ_R_*) and advancing angles (*θ_A_*), CAs and surface tensions (*γ*) of different DNAs were adopted as differential and correlative signals for analysis, contributing to a successful discrimination of the five kinds of DNAs and one control sample (Fig. 3N and Table S3). The 3D representation of the various analyte clusters demonstrated the similarities between data clusters. In Fig. 3O, graphical output of hierarchical clustering analysis (HCA) showed six major groups, reflecting subtle and accurate details. The dual-regulation strategy provides a potential universal sensing platform for multi-analyte discrimination.

### Molecular contact mechanism at the structural interface with micro-grooves between RCA droplets and lubricants

**Figure 4.**
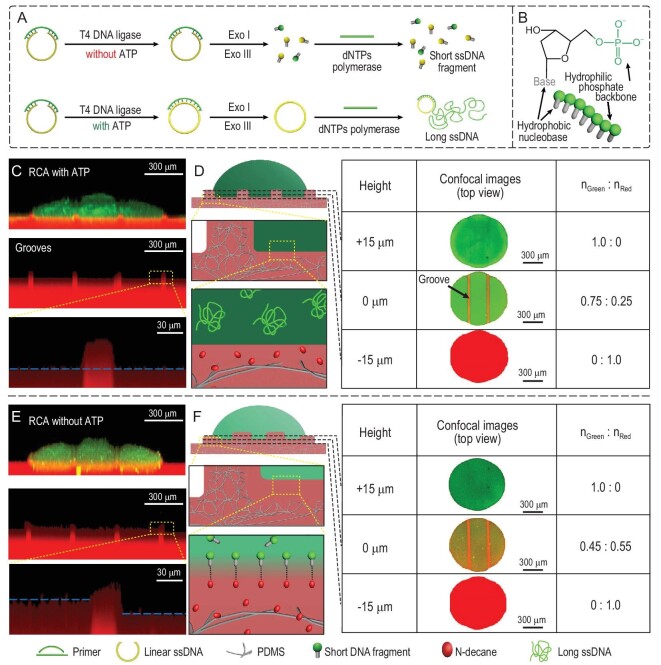
Molecular contact mechanism at the micro-groove structural interface. (A) Working principle of the ATP-triggered RCA reaction. Only in the presence of ATP, long ssDNA can be synthesized by RCA reaction. (B) Illustration of the nucleotide structure. (C) and (D) Side-view confocal image (depth scan) and schematic diagram of the RCA droplet with 40 mM ATP. Possible intermolecular hydrophobic interactions at the interface are shown on the right. (E) and (F) Side-view confocal image and schematic diagram of RCA droplet without ATP sitting on the slippery surface.

The mechanism of precise control of CSA in the liquid phase was explored. Different liquid resistance came from the different chemical constitutions in the RCA droplets with or without ATP (Fig. 4A). Without ATP, short ssDNA fragments existed in the droplet. The short ssDNA fragments consisted of hydrophilic phosphate backbones and relatively hydrophobic nucleobases (Fig. 4B), contributing to strong hydrophobic interaction with lubricant molecules [37,38]. This strong hydrophobic interaction hindered the RCA droplet from sliding on the substrate. For the RCA droplet with ATP, ssDNA with average long chains was generated (Fig. S8). Because of the long-chain ssDNA’s base–base stacking and reduced flexibility, weak hydrophobic interaction with lubricant molecules was formed. The weak hydrophobic interaction induced the RCA droplet to slide easily.

In our experiment, although the CAs were almost the same for the RCA droplets with different ATP concentration sitting on the substrates, the heights of the droplet/lubricant interfaces were various (Table S4). Confocal laser scanning microscopy in scan mode was employed to investigate the hydrophobic interaction between RCA droplets and the lubricant-infused surface with periodic micro-grooves (Fig. 4C–F). The confocal images in the green channel recorded the RCA droplet, with *n_green_* representing the proportion of the droplet. The confocal images in the red channel recorded the lubricant, with *n_red_* representing the proportion of the lubricant. After removing the green channel, droplet/lubricant interfaces of RCA droplets with or without ATP were observed. For the RCA droplet with ATP, the interfacial heights of the droplet/lubricant and air/lubricant were the same (blue dashed line in Fig. 4C). However, for the droplet without ATP, the interfacial height of the droplet/lubricant was higher than that of the air/lubricant (blue dashed line in Fig. 4E). This higher droplet/lubricant interface demonstrated that the lubricant was squeezed into the RCA droplet, indicating a strong hydrophobic interaction. To further demonstrate the strong hydrophobic interaction existing in the droplet without ATP, distributions of droplet/lubricant in different surfaces were analysed through the value of *n_green_*/*n_red_* (Fig. 4D and F). The horizontal surface at the half height of the micro-grooves was defined as the base surface (0 μm). Due to the height of the micro-grooves after swelling being ∼30 μm, the base surface was at a height of 15 μm. At the base surface, *n_red_* was ∼25% and ∼55% for RCA droplets with ATP and without ATP, respectively. On the surfaces that were 15 μm higher (+15 μm) and 15 μm lower (–15 μm) than the base surface, the distributions of droplet/lubricant for droplets with ATP and without ATP were the same. Therefore, the higher lubricant ratio at the base surface for a droplet without ATP suggested that the lubricant was squeezed into the droplet. The droplet/lubricant interface on a tilted surface was also characterized by an inverted fluorescence microscope (Videos S1 and S2). Compared to the RCA droplet without ATP, conspicuous pitting damage occurred on the interface between the lubricant and the droplet. What is more, compared to the substrate without micro-grooves in our previous research, the micro-groove structural substrate provided a droplet/lubricant interface with a much larger area, contributing to a reasonably stronger hydrophobic interaction (Figs S9 and S10).

### Mechanism of the orthogonal dual-regulation strategy on lubricant-infused substrate structure with periodic micro-grooves

To demonstrate that RCA droplets with a small change in the ATP concentration can be distinguished in the 90° sliding direction, three different methods, including confirmation of orthogonal resistance from liquid and solid phases, were used to calculate CSAs based on Dussan's model [39] and force investigation of RCA droplets was adopted to explore the mechanism of the dual-regulation strategy.

#### Confirmation of orthogonal resistance from liquid and solid phases

First, hydrophobic interaction from liquid phase was assumed as orthogonal to resistance from solid phase. In this experiment, when the RCA droplet with a 40-mM ATP slid in a direction of 0°, the RCA droplet suffered the lowest solid resistance (mainly dependent on the surface retention) and the lowest liquid resistance (mainly dependent on the hydrophobic interaction). Therefore, the RCA droplet in this state was considered as the ‘ground state’ in this article. Compared with the ground state, if the additional liquid resistance mainly depended on a target's concentration instead of sliding direction, and the additional solid resistance mainly depended on the sliding direction instead of the target's concentration, we considered the hydrophobic interaction from the lubricant and the anisotropic resistance from the substrate orthogonal to each other. More details about proof of the orthogonality of the hydrophobic interaction and anisotropic resistance are provided in Supporting Information.

**Figure 5.**
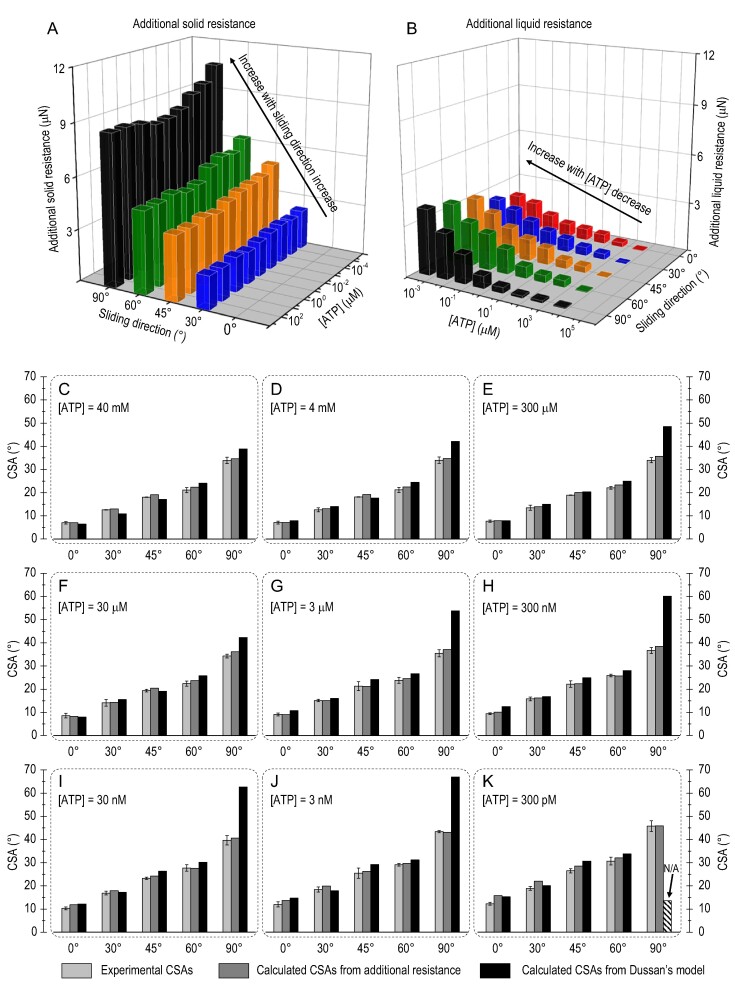
Experimental CSAs, calculated CSAs from additional resistance and calculated CSAs from Dussan's model of RCA droplets with various concentrations and different sliding directions. (A) Analysis of additional solid resistance. Compared to RCA droplets sliding in a 0° direction, various additional solid resistances of RCA droplets were suffered in the sliding directions of 30°, 45°, 60° and 90°. The additional solid resistance comes from surface retention. (B) Analysis of additional liquid resistance. Compared to RCA droplets with 40 mM ATP, various additional liquid resistances of RCA droplets were suffered with ATP concentrations of 4 mM, 300 μM, 30 μM, 3 μM, 300 nM, 30 nM, 3 nM and 300 pM. The additional liquid resistance comes from hydrophobic interaction. (C) 40 mM, (D) 4 mM, (E) 300 μM, (F) 30 μM, (G) 3 μM, (H) 300 nM, (I) 30 nM, (J) 3 nM and (K) 300 pM. Except for the 90° sliding direction, most of the calculated CSAs from Dussan's model were well matched with the corresponding experimental CSAs.

For RCA droplets sliding in 30°, 45°, 60° and 90° directions or/and containing lower concentrations of ATP, they suffered additional resistance from solid surface or/and liquid phase. Table S5 displays the total resistance of the RCA droplets with various ATP concentrations sliding in five different directions based on experimental CSAs. For RCA droplets with the same ATP concentration but sliding in different directions, the average additional solid resistance was calculated by comparing the resistance between 0° and other sliding directions (Fig. 5A and Table S6): 2.1 ± 0.2, 4.0 ± 0.4, 5.2 ± 0.5 and 9.0 ± 0.8 μN for sliding directions of 30°, 45°, 60° and 90°, respectively. This result demonstrates that the solid resistance mainly depends on the RCA droplet's sliding direction. Meanwhile, for RCA droplets sliding in the same directions but with different ATP concentrations, the average additional liquid resistance can be estimated by comparing the resistance between an ATP concentration of 40 mM and other concentrations (Fig. 5B and Table S7): 0.0 ± 0.1, 0.3 ± 0.1, 0.4 ± 0.2, 0.7 ± 0.2, 1.1 ± 0.3, 1.7 ± 0.3, 2.3 ± 0.4 and 3.0 ± 0.7 μN for ATP concentrations of 4 mM, 300 μM, 30 μM, 3 μM, 300 nM, 30 nM, 3 nM and 300 pM, respectively. These calculated resistances demonstrate that liquid resistance mainly depends on the RCA droplet's ATP concentration, displaying a weaker effect than solid resistance. Meanwhile, compared to the ground state, both additional solid and additional liquid resistance contribute to increased CSAs of RCA droplets. Therefore, calculated CSAs can be estimated by comparing the total additional resistance to the ground state:

(1) \begin{eqnarray*} && mg\ {\rm{sin}}\ \textit{CSA}{_{Direction,\quad {\rm{\ }}\left[ {ATP} \right]}} - mg\sin\ {\textit{CSA}_{groud{\rm{\ }}state}}\nonumber\\ && \quad = {\rm{\ }}\Delta f\ = \Delta {f_s}{\rm{\ }} + {\rm{\ }}\Delta {f_L}. \end{eqnarray*}

The calculated CSAs can be expressed from Equation 1:

(2) \begin{eqnarray*} && {\textit{CSA}_{{\rm{\ }}Direction,\ }} \nonumber\\ && \left[ {ATP} \right] = {\sin ^{ - 1}}\left( {\frac{{\Delta f}}{{V\bar{\rho }g}} + \sin {\textit{CSA}_{groud\ state}}_{\rm{\ }}} \right) .\nonumber\\ \end{eqnarray*}

In Equation 1, *Δf* is the total additional resistance, which equals the average additional solid resistance (Table S6) added to the average additional liquid resistance (Table S7). In Equation 2, *V* is the volume of the droplet, *g* is the gravitational acceleration and $\bar{\rho }$ is the average density of the RCA with various nine concentrations. For example, compared with the ground state, an RCA droplet sliding in a direction of 60° with an ATP concentration of 300 nM would suffer ∼6.3 μN of additional resistance, including 5.2 μN of additional solid resistance and 1.1 μN of additional liquid resistance. This 6.3-μN additional resistance results in the calculated CSA increasing to 25.7° (the corresponding experimental CSA of 25.9 ± 0.6°). The calculated CSAs (Fig. 5C–K and Table S8) based on additional resistance are well matched with the experimental CSAs, with most calculated deviations <10% (Table S9). However, for RCA droplets with an ATP of 30 nM, 3 nM and 300 pM sliding in a 0° direction, their calculated CSAs demonstrated deviations of >10%. The relatively small experimental CSAs (all <15°) may be the potential reason. To sum up, we considered that the solid resistance adjusted by the droplets’ sliding direction was orthogonal to the liquid resistance manipulated by the RCA droplets’ ATP concentration. Both the solid resistance and the liquid resistance contributed to the precise control of RCA droplets’ motion behavior and sensitive sensing.

#### Calculated CSAs based on Dussan's model

In order to further confirm the mechanism of the dual-regulation strategy, comparison of calculated CSAs based on Dussan's model with the corresponding experimental CSAs was also adopted. The calculated CSAs based on Dussan's model can be described as:

(3) \begin{eqnarray*} {\left( {\frac{{\rho g\sin \textit{CSA}}}{\gamma }} \right)^{3/2}} V = {\left( {\frac{{96}}{\pi }} \right)^{1/2}}\frac{{{{(\cos {\theta _R} - \cos {\theta _A})}^{3/2}}{{(1 + \cos {\theta _A})}^{3/4}}\left( {1 - \frac{3}{2}\cos {\theta _A} + \frac{1}{2}{{\cos }^3}{\theta _A}} \right)}}{{{{(\cos {\theta _A} + 2)}^{3/2}}{{(1 - \cos {\theta _A})}^{9/4}}}}.\quad\quad\quad \end{eqnarray*}

Experimental CSAs, calculated CSAs from additional resistance and calculated CSAs from Dussan's model of RCA droplets with various concentrations and sliding in five directions are displayed in Fig. 5. Except for the 90° sliding direction, most of the calculated CSAs from Dussan's theory were well matched with the corresponding experimental CSAs, with deviation of <20% (Fig. S11). Most obviously, deviation for RCA droplets in the 90° sliding direction may due to unsatisfied precondition of Dussan's model: CA hysteresis should be <10° [39]. However, in our experiment, most of the CA hysteresis values were <10°, except in the cases of the 90° sliding direction (Table S10). For example, for a 3-nM ATP droplet, the CA hysteresis values were 4.5 ± 0.3°, 5.4 ± 0.3°, 8.0 ± 0.3°, 8.6 ± 0.3° and 15.2 ± 0.2° for sliding directions of 0°, 30°, 45°, 60° and 90°, respectively. Specifically, for an RCA droplet with an ATP concentration of 300 pM sliding in a 90° direction (Fig. 5K), the calculated CSA is inapplicable for Dussan's model because of the value of sin*CSA* being >1 in Equation 3. The calculated CSAs from Dussan's model further demonstrated the precise control of RCA droplets’ motion behaviors and the reliability of CSA-based sensitive detection through a dual-regulation strategy.

#### Force investigation of RCA droplets in 0° and 90° sliding directions

To demonstrate that RCA droplets with a small change in ATP concentrations can be better distinguished in the 90° sliding direction than other sliding directions, force investigation of RCA droplets on a tilted periodic micro-grooves slippery surface was adopted. For a droplet to move on a surface tilted at an angle *α*, the tangential component of the gravitational force, *mg*sin*α*, acting on the droplet has to exceed the resistance, *f*. The work done by gravity is equal to the work done by the surface tension in wetting a unit area of the surface [40]:

(4) \begin{equation*} mg\sin \alpha = \gamma D\left( {\cos {\theta _R} - \cos {\theta _A}} \right). \end{equation*}

*m*, *γ* and *D* are the mass, surface tension and wetting width of the droplet, respectively; *g* is the acceleration due to gravity. When the droplet is just about to move, *α* is equal to CSA:

(5) \begin{equation*} mg\sin \textit{CSA} = \gamma D\left( {\cos {\theta _R} - \cos {\theta _A}} \right). \end{equation*}

Table S5 represents the relationships between the ATP concentration, the droplet sliding direction and the resistance. For RCA droplets with the same sliding direction, the resistance increased with the decrease in ATP concentration. In this case, the resistance depended on interfacial hydrophobic interaction from the liquid phase. Meanwhile, for RCA droplets with the same ATP concentration, the resistance increased when the droplet's sliding direction changed from 0°, 30°, 45°, 60° to 90°. Comparing with the 0° sliding direction, the RCA droplet suffered additional resistance from the micro-grooves and displayed increased CA hysteresis in the other four sliding directions [41]. This phenomenon contributed that RCA droplets with a small change in ATP concentrations can be better distinguished in a sliding angle of 90° than in a sliding direction of 0°.

To demonstrate this speculation, we investigated the resistance of two RCA droplets with a small difference in ATP concentrations in a sliding direction of 0° and 90°, respectively (Fig. S12A). When the RCA droplets were sliding in a direction of 0°, the resistance mainly depended on the liquid-phase resistance *f_L_*(C) (Fig. S12B and C). In this case, *f_L_*(C) comes from the hydrophobic interaction at the droplet/lubricant interface, which increased when the ATP concentration decreased. Therefore, according to Equation 5, when two RCA droplets are sliding in a 0° direction, their difference in resistance can be expressed as:

(6) \begin{eqnarray*} &&{f_2} - {f_1} = {f_L}( {{C_2}} )\! -\! {f_L}( {{C_1}} ) \nonumber\\ &&\quad = mg( {\sin CS{A_2} - \sin CS{A_1}} ) \nonumber\\ &&\quad = {\gamma _2}{D_{0^\circ }}( {\cos {\theta _{R2}} - \cos {\theta _{A2}}} )\nonumber\\ &&\qquad -\, {\gamma _1}{D_{0^\circ }}( {\cos {\theta _{R1}} - \cos {\theta _{A1}}} ). \end{eqnarray*}

When RCA droplets were sliding in a 0° direction, there was no obvious CA hysteresis. For example, when *C*_1_ was 300 nM, *C*_2_ was 30 nM, *θ_R_*_1_ = 45.2 ± 0.7°, *θ_A_*_1_ = 48.9 ± 0.1°, *θ_R_*_2_ = 44.2 ± 0.3°, *θ_A_*_2_ = 47.7 ± 0.2°; *γ*_1_ = 34.14 mN/m; *γ*_2_ = 34.48 mN/m (Fig. S13); *D*_0°_ = 3024 μm; *D*_90°_ = 3000 μm. Based on calculation, we considered *γ*_1_ ≈ *γ*_2_ ≈ *γ*; *D*_0°_ ≈ *D*_90°_ ≈ *D*. Therefore, Equation 6 can be represented as:

(7) \begin{eqnarray*} &&{f_2} - {f_1}_{} = {f_L}( {{C_2}} ) - {f_L}( {{C_1}} ) \nonumber\\ &&\quad = {\rm{ }}mg( {\sin CS{A_2} - \sin CS{A_1}} )\nonumber\\ &&\quad = {\rm{ }}0.044{\gamma _2}{D_{0^\circ }} - 0.048{\gamma _1}{D_{0^\circ }} \approx {\rm{ }}0.004\gamma D.\nonumber\\ \end{eqnarray*}

On the other hand, when the RCA droplets were sliding in a direction of 90°, the resistance not only came from the liquid phase *f_L_*(*C*), but also relied on the solid phase *f_S_*(*SD*, C) due to the energy barrier given by the micro-grooves (Fig. S12D and E). The solid-phase resistance depended on the sliding direction. Therefore, when two RCA droplets are sliding in a 90° direction, their difference in resistance can be expressed as:

(8) \begin{eqnarray*} &&{f_2}^{\prime} - {f_1}^{\prime} = {\rm{ }}mg{( {\sin \textit{CSA}{_2}{'} - \sin \textit{CSA}{_1}{'}} )_{}}\nonumber\\ &&\quad = {f_L}( {{C_2}} ){\rm{ }} - {f_L}( {{C_1}} ){\rm{ }}\nonumber\\ &&\qquad + {f_S}( {S{D_{90}},{C_2}} ){\rm{ }} - {f_S}( {S{D_{90}},{C_1}} )\nonumber\\ &&\quad = {\gamma _2}{D_{90^\circ }}( {\cos {\theta _{R2}}{'} - {\rm{ }}\cos {\theta _{A2}}{'}} ){\rm{ }}\nonumber\\ &&\qquad -\, {\gamma _1}{D_{90^\circ }}( {\cos {\theta _{R1}}{'} - {\rm{ }}\cos {\theta _{A1}}{'}} ). \end{eqnarray*}

Based on the photo of RCA droplets sliding in a 90° direction, obvious CA hysteresis values were observed: *θ_R_*_1_^′^ = 42.2 ± 0.6°, *θ_A_*_1_^′^ = 56.9 ± 0.8°, *θ_R_*_2_^′^ = 44.5 ± 0.9°, *θ_A_*_1_^′^ = 59.4 ± 1.0° (Table S11). Therefore, Equation 8 can be represented as:

(9) \begin{eqnarray*} &&{f_2}^{\prime} - {f_1}^{\prime} = {\rm{ }}mg{( {\sin \textit{CSA}{_2}\text{'} - \sin \textit{CSA}{_1}\text{'}} )_{}} \nonumber\\ &&\quad = {f_L}( {{C_2}} ){\rm{ }} - {f_L}( {{C_1}} ){\rm{ }} + {f_S}( {S{D_{90}},{C_2}} ){\rm{ }}\nonumber\\ &&\qquad -\, {f_S}( {S{D_{90}},{C_1}} ) = {\rm{ }}0.203{\gamma _2}{D_{90^\circ }}\nonumber\\ &&\qquad -\, {\rm{ }}0.195{\gamma _1}{D_{90^\circ }} \approx {\rm{ }}0.08\gamma D. \end{eqnarray*}

Comparing Equations 7 and 9, when these two RCA droplets are sliding in a 90° direction, the additional resistance from the solid phase strengthens their difference in resistance by ∼20-fold of that in a 0° direction. Therefore, for RCA droplets with a small difference in ATP concentrations, their amplified difference in resistance further enhanced their difference in CSAs: sin*CSA*_2_ – sin*CSA*_1_ < sin*CSA*_2_^′^ – sin*CSA*_1_^′^; *CSA*_2_* – CSA*_1_ < *CSA*_2_^′^ – *CSA*_1_^′^.

According to the experimental *CSA*_2_ = 9.4 ± 0.4° ([ATP] = 300 nM; 0°), *CSA*_1_ = 10.3 ± 0.6° ([ATP] = 30 nM; 0°), *CSA*_2_^′^ = 36.7 ± 1.3° ([ATP] = 300 nM; 90°) and *CSA*_1_^′^ = 39.7 ± 2.0° ([ATP] = 30 nM; 90°), the difference between *CSA*_2_ and *CSA*_1_ was smaller than the difference between *CSA*_2_^′^ and *CSA*_1_^′^. Therefore, due to the obvious CA hysteresis and additional solid-phase resistance, RCA droplets with a small change in ATP concentration can be better distinguished in a 90° sliding direction.

### Universal biosensing applications: sensitive detection of miRNAs, thrombin and kanamycin

**Figure 6.**
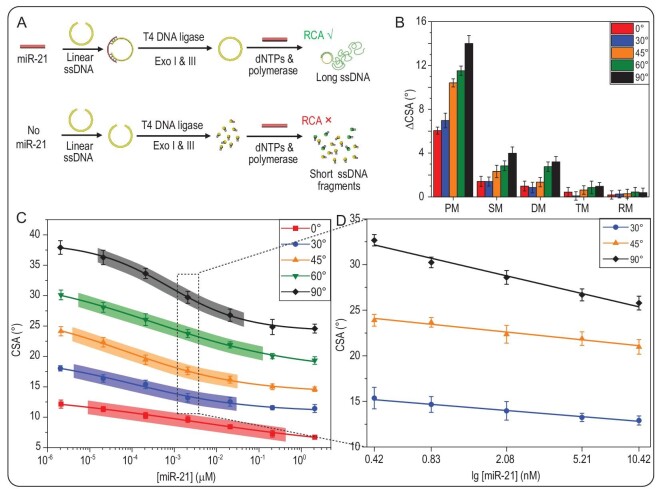
Droplet motion applied for miR-21 detection. (A) Working principle of detecting miR-21 based on RCA droplet's motion behaviors. (B) Specificity investigations for PM, SM, DM, TM and RM miR-21. (C) CSAs of RCA droplets with various miR-21 concentrations (2.08 pM, 20.8 pM, 208 pM, 2.08 nM, 20.8 nM, 208 nM and 2.08 μM) in five different sliding directions, respectively. The colorful shadows are the dynamic ranges of detection miR-21 in different sliding directions. (D) In sliding directions of 45°, 60° and 90°, RCA droplets with miR-21 concentrations of 0.41, 0.83, 2.08, 5.21 and 10.42 nM can be sensitively detected.

To demonstrate that this sensitive detection method is universally applicable to a broad variety of targets, a serial of RCA droplets that contained important biomarkers with various concentrations were tested. Herein, miRNAs [42–45], thrombin [46–48] and kanamycin [49,50] were selected as biomarkers due to their key roles in life processes. With the absence of miR-21, thrombin or kanamycin, the RCA reaction cannot be triggered, leading to massive short ssDNA fragments existing in the droplet (Fig. 6A). The strong interfacial interaction between short ssDNA fragments and lubricant would hinder the droplets from sliding. However, the circular DNA could be formed in the presence of miR-21, thrombin or kanamycin, resulting in the process of RCA and generating long ssDNA. Thus, there was a weak interfacial interaction at the droplet/lubricant interface, enabling the droplet to slide easily (Figs 6A, S14A and S15A). As shown in Fig. 6, the CSA decreased when the miR-21 concentration increased from 2.08 pM to 2.08 μM in five sliding directions. Various dynamic ranges and sensitivity were realized in different sliding directions (Fig. 6C and Table S2). In the sliding direction of 0°, the widest sensing dynamic range, 4.77 × 10^4^-fold with miR-21 concentration between 9.26 × 10^–6^ and 4.42 × 10^–1^ μM can be achieved. Due to the greater sensitivity in sliding directions of 30°, 45° and 90°, a series of miRNA droplets with smaller change in miR-21 concentration, including 416 pM, 832 pM, 2.08 nM, 5.21 nM and 10.42 nM, can be distinguished (Fig. 6D). In addition, the specificity of miR-21 was also demonstrated by miR-21 analogs, including single-base-mismatched (SM) miR-21, double-base-mismatched (DM) miR-21, triple-base-mismatched (TM) miR-21 and random matched RNA (RM), were added (Fig. 6B). The above results illustrate that the detection based on dual-regulation strategy is suitable for sensitive detections of miR-21. In addition, thrombin and kanamycin as detection targets are also realized (Figs S14 and S15).

## DISCUSSION

In this research, a dual strategy for biomarkers’ sensitive detection with adjustable dynamic ranges was developed. Through adjusting the resistance met by RCA droplets from liquid and solid phases, their motion behaviors can be precisely controlled. In detail, by adjusting targets’ concentrations and RCA droplets’ sliding directions on the substrate, the hydrophobic interaction from the liquid phase and anisotropic resistance from the solid phase can be finely tuned, respectively. The hydrophobic interaction is considered to be orthogonal to anisotropic resistance, which contributes to the effective control of bio-droplets’ motion behavior and tunable sensitivity with relative dynamic range. Specifically, when RCA droplets slide in the 90° direction, they would suffer the largest additional resistance from the micro-grooves and displayed obvious CA hysteresis. The great CA hysteresis contributes to an optimized sensitivity for distinguishing RCA droplets with a 2-fold change in targets’ concentration, within their corresponding dynamic range. Meanwhile, in a direction of 0°, a maximum dynamic range of 10^6^-fold also can be achieved.

To demonstrate the dual strategy's ability of precise control of droplets’ motion, two methods were adopted to estimate CSAs. Calculated CSAs based on analysing additional solid and liquid resistance, as well as theoretical CSAs from Dussan's model were included and compared with experimental CSAs. For calculated CSAs based on force investigation, they were well matched with the experimental CSAs of RCA droplets with ATP concentrations of >300 nM and sliding in directions of 45°, 60° and 90°. Although these calculated CSAs display minor deviation from experimental CSAs, a significant amount of experimental data are required for analysing resistance. Dozens of experiments should be performed to confirm a single resistance for a specific case. On the other hand, to achieve calculated CSAs from Dussan's model, only several readily accessible parameters, including density, volume, surface's tension, receding angle and advancing angle of the droplet, are needed. Based on Dussan's model, most of the theoretical CSAs matched nicely with the corresponding experimental CSAs, except the RCA droplets sliding in a 90° direction. In these cases, CA hysteresis values were >10°, which did not satisfy the precondition of Dussan's model. Thus, an accurate, effective and feasible theoretical model is still expected to realize accurate estimation of droplet CSAs. Based on an accurate theoretical model, we hope that other researchers may be inspired to rationally design sensitive detection with tunable dynamic ranges for various bio-targets. Meanwhile, this method also realized sensitive detection of ATP, miRNA, thrombin, kanamycin and recognition of five kinds of DNA. This orthogonal dual-regulation strategy for sensitive detection with adjustable dynamic range still needs further improvement. Effectively adjusting the position of the dynamic range and narrowing it to the appropriate concentration range for specific targets’ detection is also one of the important directions for future research. The sensitive detection strategy will provide new opportunities and significant insights for research on liquid-directional transportation, liquid printing, separation, microfluidic devices, biosensor and related research.

## METHODS

Methods are available at *NSR* online.

## Supplementary Material

nwac048_Supplemental_Files
