# Supplementary material for: An orthogonal dual-regulation strategy for sensitive biosensing applications
Source: Natl Sci Rev. 2022 Mar 12;9(9):nwac048. doi: 10.1093/nsr/nwac048 (PMC9584063; doi:10.1093/nsr/nwac048)
Supplement: nwac048_Supplemental_Files [file nwac048_supplemental_files.zip › Supporting_Information.docx]

**Supporting information**

**An Orthogonal Dual-Regulation Strategy for Sensitive Bio-sensing Applications**

Xian Yang^1,6,†^, Jinhua Wang^5,1,†^, Zhongfeng Gao^1,3,†^, Weiqi Zhang^1^, Hai Zhu^1^, Yongjun Song^1^, Quan Wang^1^, Yu Huang^1,2^*, Mingjie Liu^4^, Fan Xia^1,2^** and Lei Jiang^4^

1 State Key Laboratory of Biogeology and Environmental Geology, Engineering Research Center of Nano-Geomaterials of Ministry of Education, Faculty of Material Science and Chemistry, China University of Geosciences, Wuhan 430074, China

2 Zhejiang Institute, China University of Geosciences, Hangzhou, 311305, China

3 Shandong Provincial Key Laboratory of Detection Technology for Tumor Markers, College of Chemistry and Chemical Engineering, Linyi University, Linyi 276005, China

4 Key Laboratory of Bio-Inspired Smart Interfacial Science and Technology of the Ministry of Education, School of Chemistry and Environment, Beihang University, Beijing, 100191, China

5 State Key Laboratory of Proteomics, Beijing Proteome Research Center, National Center for Protein Sciences (Beijing), Beijing Institute of Lifeomics, Beijing, 102206, China

6 State Grid Integrated Energy Service Group CO. LTD., Beijing, 100052, China

† These authors contributed to this work equally

*Correspondence: yuhuang@cug.edu.cn

**Correspondence: [xiafan@cug.edu.cn](mailto:xiafan@cug.edu.cn)


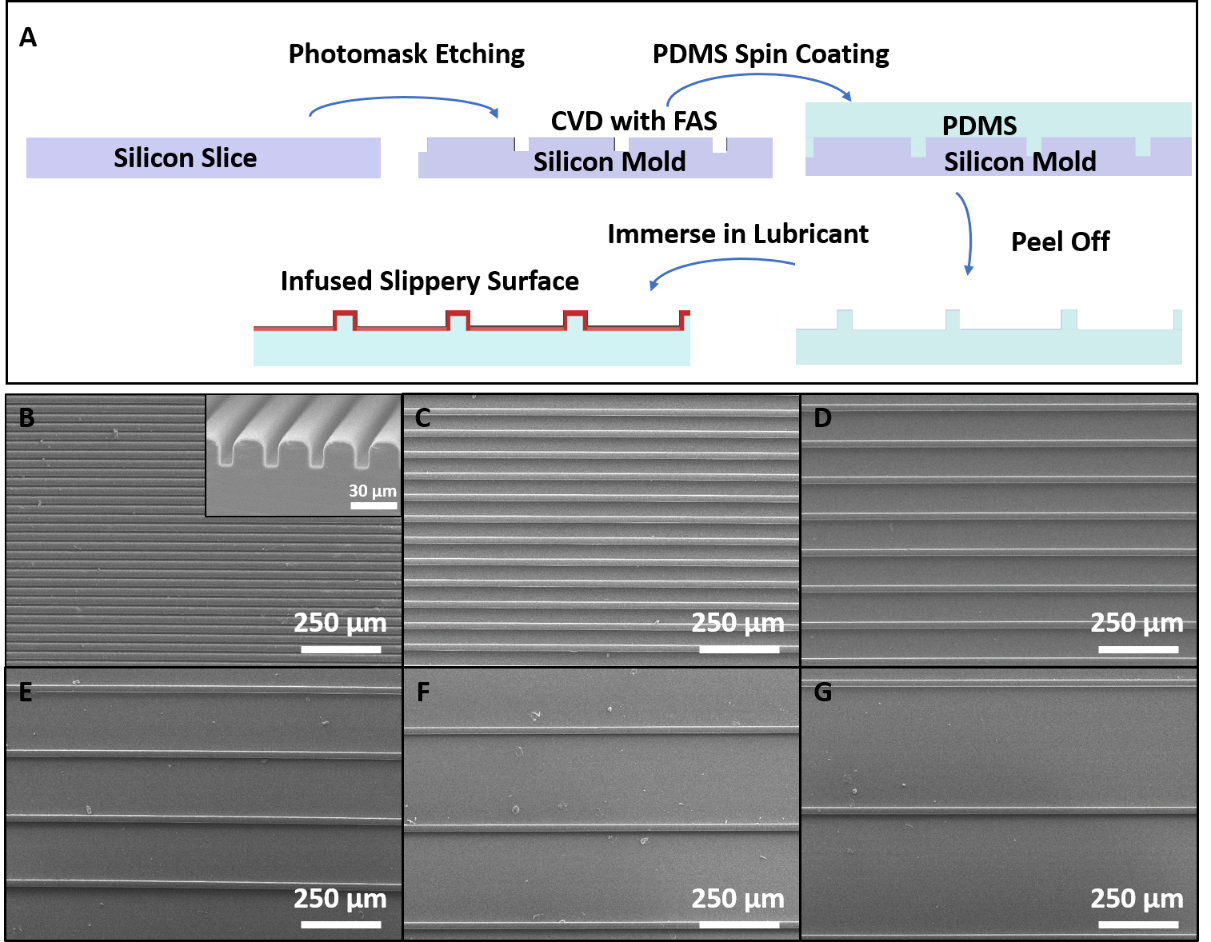


**Figure S1**. Preparation of Micro-grooves structural PDMS Slippery Surface. (A) Schematic illustration of preparation process for micro-grooves structural slippery surface, mainly consisting of three steps based on chrome plate mask photolithographic method: silicon mold preparation, polydimethylsiloxane (PDMS) replication, PDMS immersed in lubricant for fabricating lubricant-infused slippery surface. (B-G) SEM images of substrates with different morphologies. For all the ≡grooves are 20 μm in width, and 20 μm in height. (Figure S1B insert). Six different spacing specifications are designed: (B) 10 μm, (C) 50 μm, (D) 100 μm, (E) 200 μm, (F) 300 μm, (G) 400 μm.


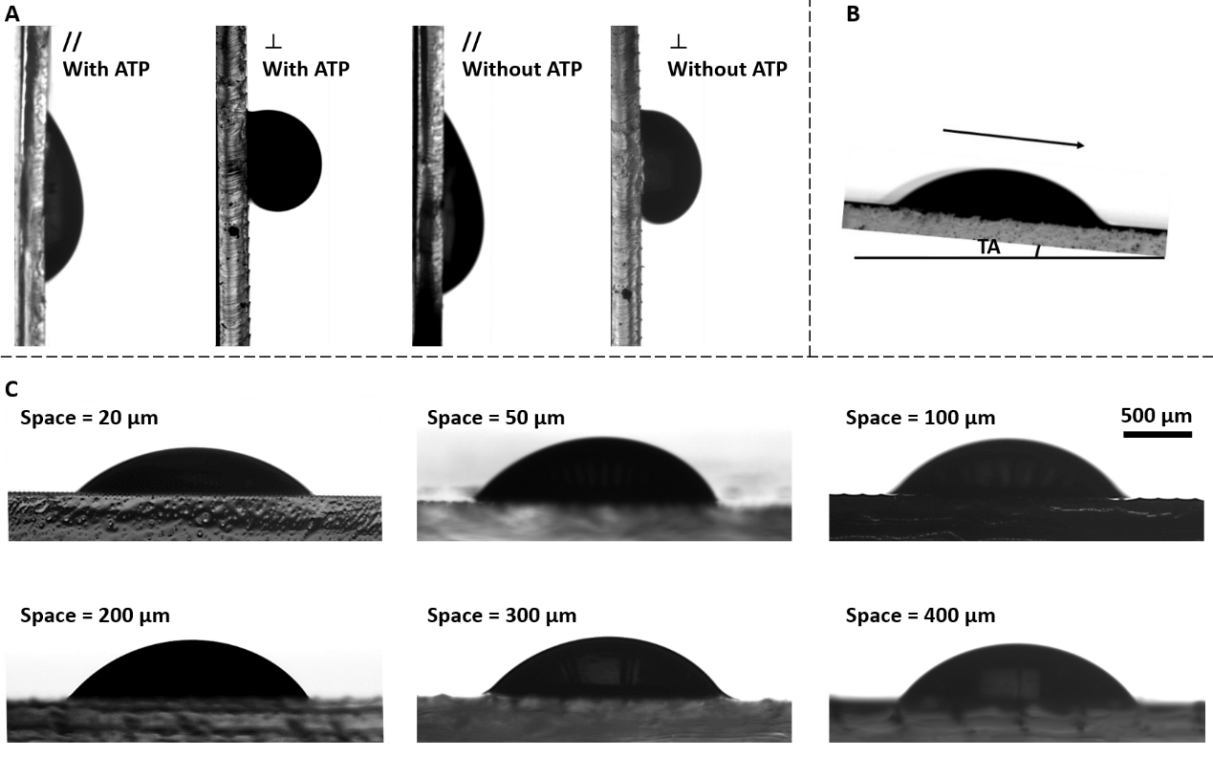
**Figure S2.** Motion behaviors of RCA droplets on anisotropic substrates. (A) RCA droplets (2 µL) with ATP (40 mM) and without ATP sitting on the micro-grooves structural PDMS substrate without lubricant infused, in 0° and 90° sliding directions. All droplets was pinned on the surface, even the surfaces were tilted to 90°. (B) Photograph of a RCA droplet (2 µL) slide easily on the lubricant-infused micro-grooves PDMS substrate in direction of 0°. TA means tilting angle in the figure. (C) Images of 2.0 μL RCA droplets siting on periodic micro-grooves surfaces with different spacing: 20 μm, 50 μm, 100 μm, 200 μm, 300 μm and 400 μm.


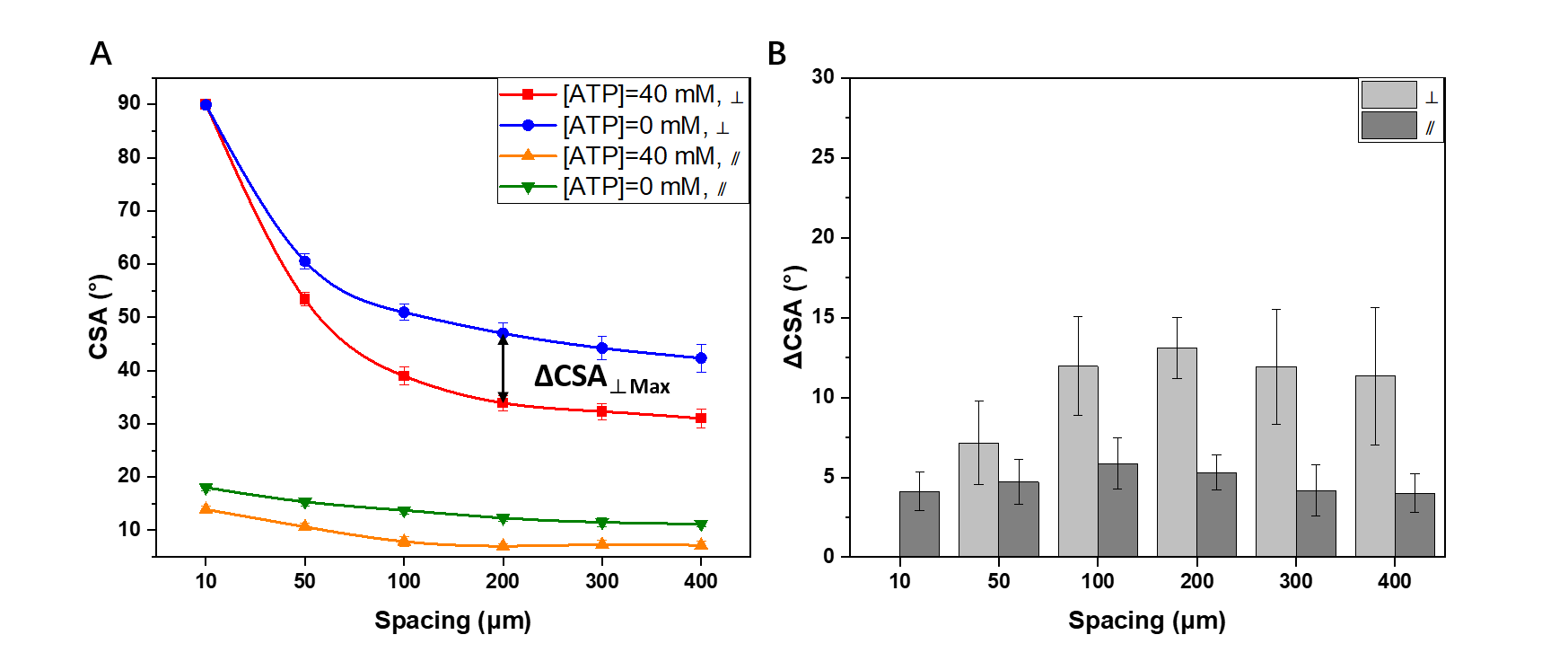
**Figure S3**. CSAs of RCA droplets on the micro-grooves structural PDMS substrates with different spacing. (A) Relationships between CSAs of RCA droplets and spacing of the micro-grooves structural PDMS. When the concentration of ATP changes from 0 to 40 mM, the biggest difference in CSAs can be realized on the substrate with spacing of 200 μm. (B) The difference in CSAs for RCA droplets without or with 40 mM ATP for substrates with different spacing. On micro-grooves structural PDMS substrates with spacing of 200 μm, the biggest difference in CSAs with stable motion behaviors were achieved. The volumes of adopted RCA droplets were all 2.0 μL.


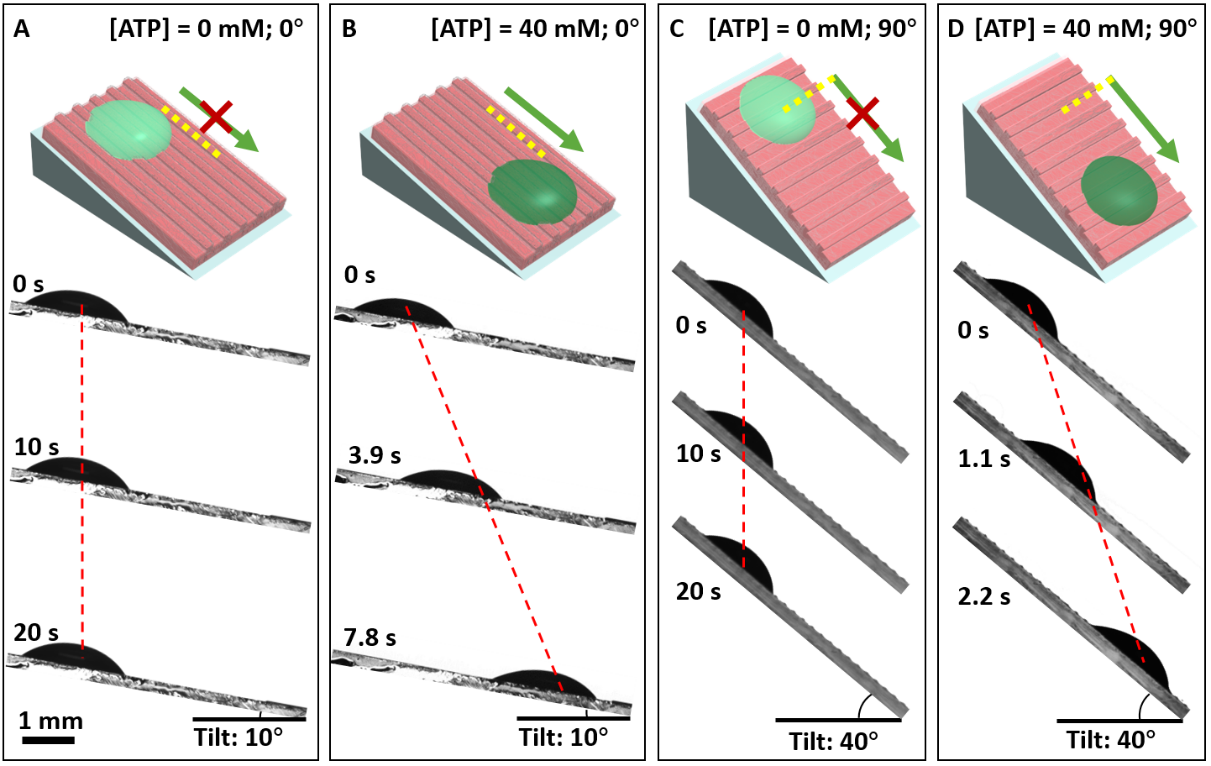
**Figure S4.** The sliding behaviors of RCA droplets on lubricant-infused PDMS substrates would change when the concentration of ATP contained in RCA droplets was different. For example, in 0° sliding direction, a RCA droplet without ATP was pinned on the periodic micro-grooves slippery surface at the tilting angle of 10°, as shown in Fig S4A. In contrast, a RCA droplet triggered by 40 mM ATP slid on the surface in 3.9s (Fig S4B). The CSAs of RCA droplets without ATP and with 40 mM ATP were 12.3 ± 1.1° and 7.0 ± 0.5° respectively. Similar phenomena were also observed for RCA droplets sliding in the 90° direction. As shown in Fig S4C- S4D, when the tilting angle of slippery surface was 40°, droplet without ATP was pinned (Fig S4C), and droplet with 40 mM ATP was sliding within 1.1s (Fig S4D). When RCA droplets were sliding in the same direction, larger CSAs were realized for RCA droplets without ATP than that of with ATP, demonstrating the regulation from liquid phase based on adjusting the ATP’s concentration was successful. On the other hand, for the RCA droplets with the same ATP concentration, their CSAs in 90° sliding direction obviously increased compared with that of in 0° sliding direction. For example, for RCA droplets with 40 mM ATP, the CSA increased from 7.0 ± 0.5° to 33.9 ± 1.4° when the sliding direction changed from 0° to 90° (Fig 2A). The results suggested the regulation from solid phase based on tuning the sliding directions was realized. The successful dual-regulation from liquid and solid phases for precise control of RCA droplets’ motion behaviors contributes to the ATP detection with tunable sensitivity and adjustable dynamic ranges.


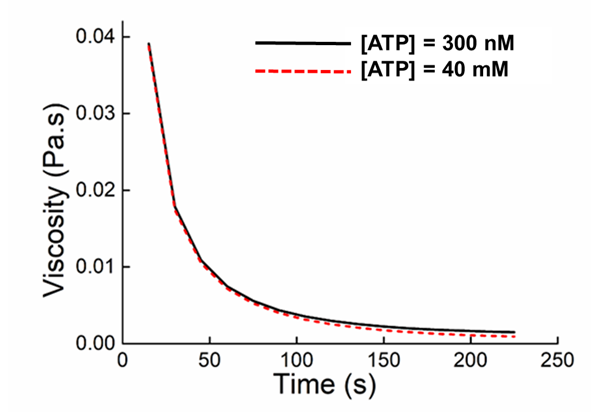
**Figure S5.** Viscosity of RCA droplets with ATP concentration of 300 nM and 40 mM. There was subtle change in viscosity of as-prepared RCA droplets.


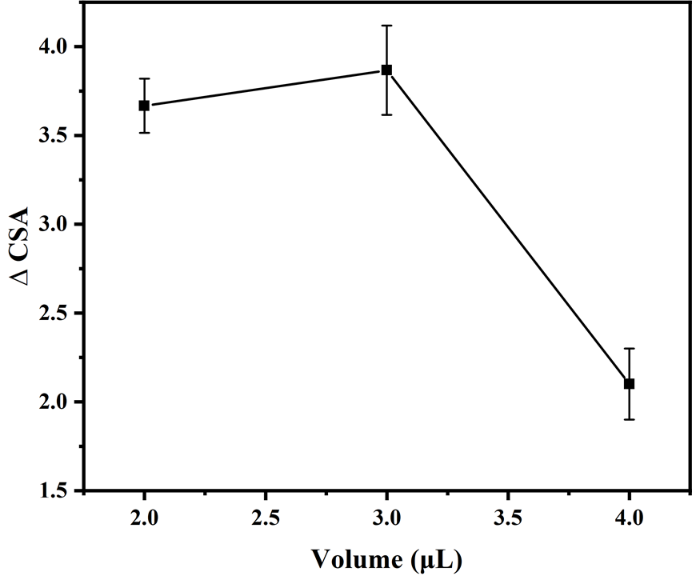


**Figure S6**. The optimal experiment of the droplet volume on micro-grooves structural substrate with lubricant-infused. The ΔCSA represents the difference in CSA of RCA droplets without ATP or with 40 mM ATP.


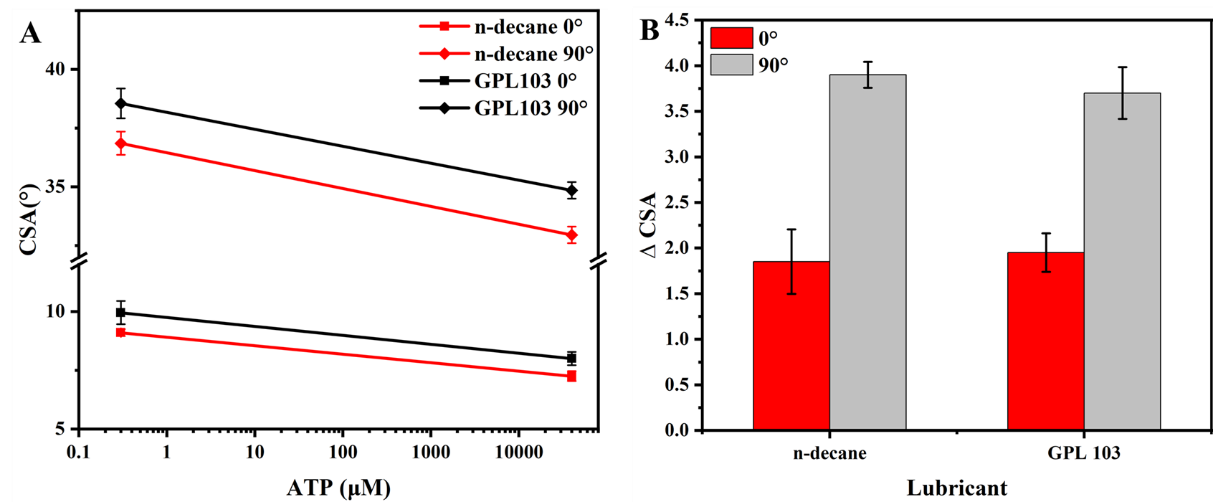


**Figure S7.** On micro-groove structural substrates infused with n-decane and GPL 103, (A) the relationship between CSAs and RCA droplets (ATP concentrations of 300 nM and 40 mM) with sliding directions of 0° and 90°, respectively, and (B) change in CSAs (ΔCSA) of RCA droplets (ATP concentrations of 300 nM and 40 mM) with sliding directions of 0° and 90°, respectively. For substrates infused with n-decane and GPL 103, the corresponding ΔCSA of RCA droplets with sliding directions of 90° are greater than that with the sliding directions of 0°.


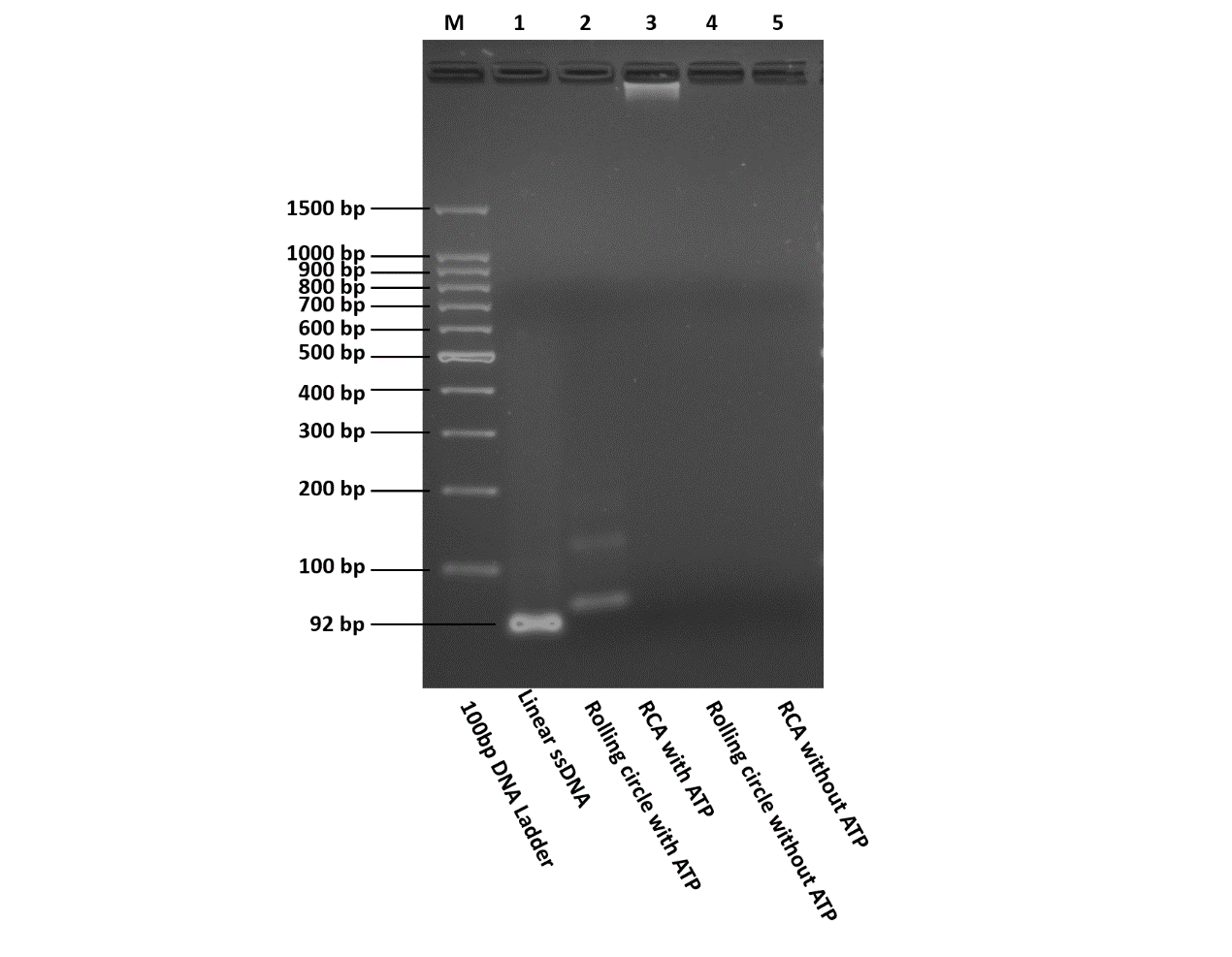
**Figure S8**. Gel electrophoresis images of the droplet experienced RCA reaction for 3 h. The result showed that RCA reaction can be triggered with ATP. (M) 100 bp DNA size marker; (1) 1 μM linear ssDNA with 92 bases; (2) linear ssDNA rolling circle with ATP; (3) RCA reaction with ATP; (4) linear ssDNA rolling circle without ATP; (5) RCA reaction without ATP. The concentration of ATP was 40 mM.


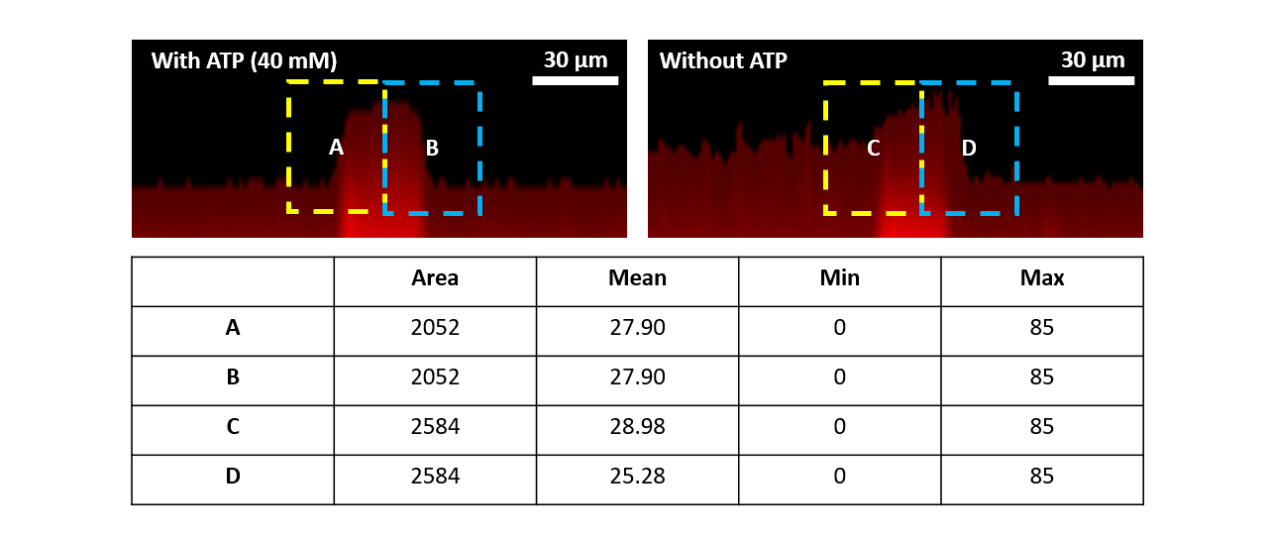
**Figure S9**. Calculation of [fluorescence](javascript:;) of droplet/lubricant interface with ATP and without ATP by Image J software. A, B, C and D were all the selected area. A and C were droplet/lubricant interface, displaying the lubricant in RCA droplet. B and D were air/lubricant interface, displaying the lubricant outside RCA droplet. The minus signal of backgrounds all were 0, the maximum signal of backgrounds all were 85. In the case of the droplet with ATP, the height of red lubricant in droplet/lubricant interface was as high as the interface of air/lubricant. In the case of the droplet without ATP, the height of red lubricant in droplet/lubricant interface was higher than that of air/lubricant.


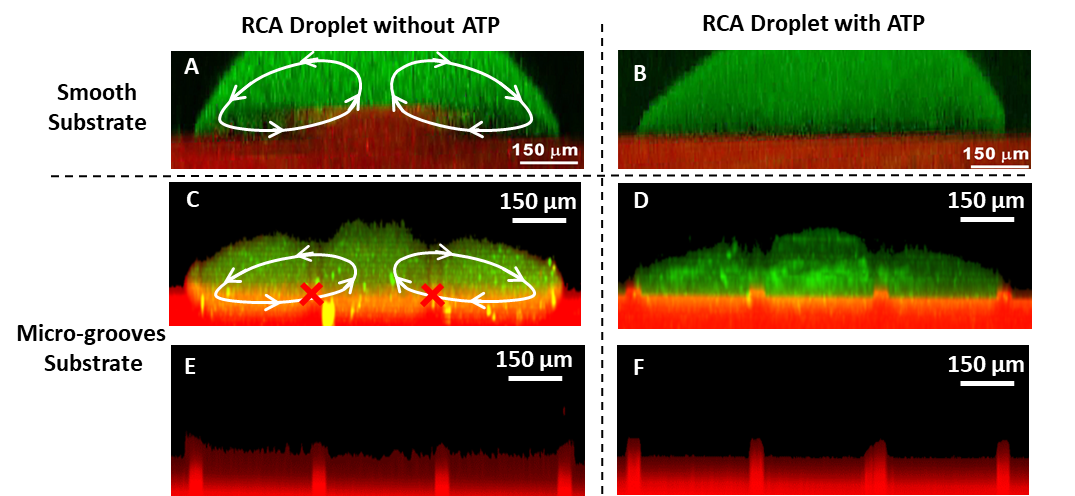
**Figure S10.** Side view confocal images (depth scan) of the RCA droplet without or with ATP (40 mM) on lubricant-infused smooth substrate and micro-grooves structural substrate, displaying different droplet/lubricant interfaces. The RCA droplet and lubricant are two kinds of solution with different surface tensions, which would induce Marangoni effect [1]. (A, B) When RCA droplet with ATP sitting on lubricant-infused smooth surface, the droplet/lubricant interface in the center of the droplet was squeezed into the droplet [2]. We consider this phenomenon may come from the small lubricant droplets converged to the center of droplet under the action of the transverse flow in the water phase [3]. The white circular arrow indicates the direction of liquid flow under the Marangoni effect. (C, D) On the lubricant-infused micro-grooves structural substrate, the transverse flow inside the droplet was blocked by the micro-grooves. Meanwhile, the micro-grooves structural substrate provided much larger area of droplet/lubricant interface. As a result, the whole droplet/lubricant interface was lift steadily for RCA droplet without ATP sitting on micro-groove structural substrate. (E, F) The corresponding confocal images in red channel of C and D, displaying the lifting of droplet/lubricant interface of RCA droplet without ATP.


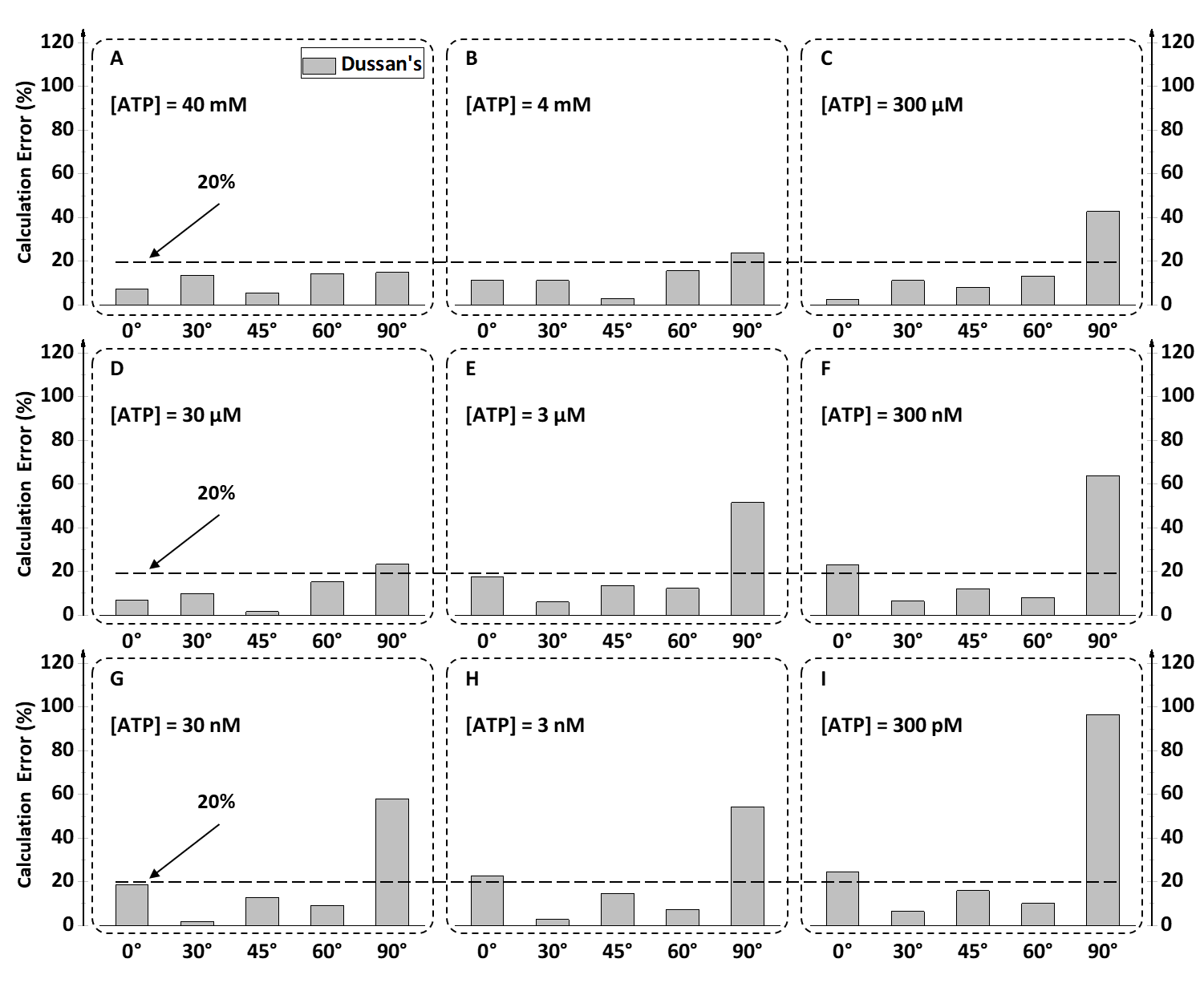
**Figure S11**. Calculation errors based on comparing experimental CSAs and calculated CSAs from Dussan’s model in various sliding directions. Most of the calculated CAs from Dussan’s theory are well matched the experimental CAs, except in 90° sliding direction. The obviously deviation in 90° sliding direction may resulted from against the precondition of Dussan’s model: the CA hysteresis should be less than 10°. In the experiment, most of the CA hysteresis in the 90° direction were more than 10°.


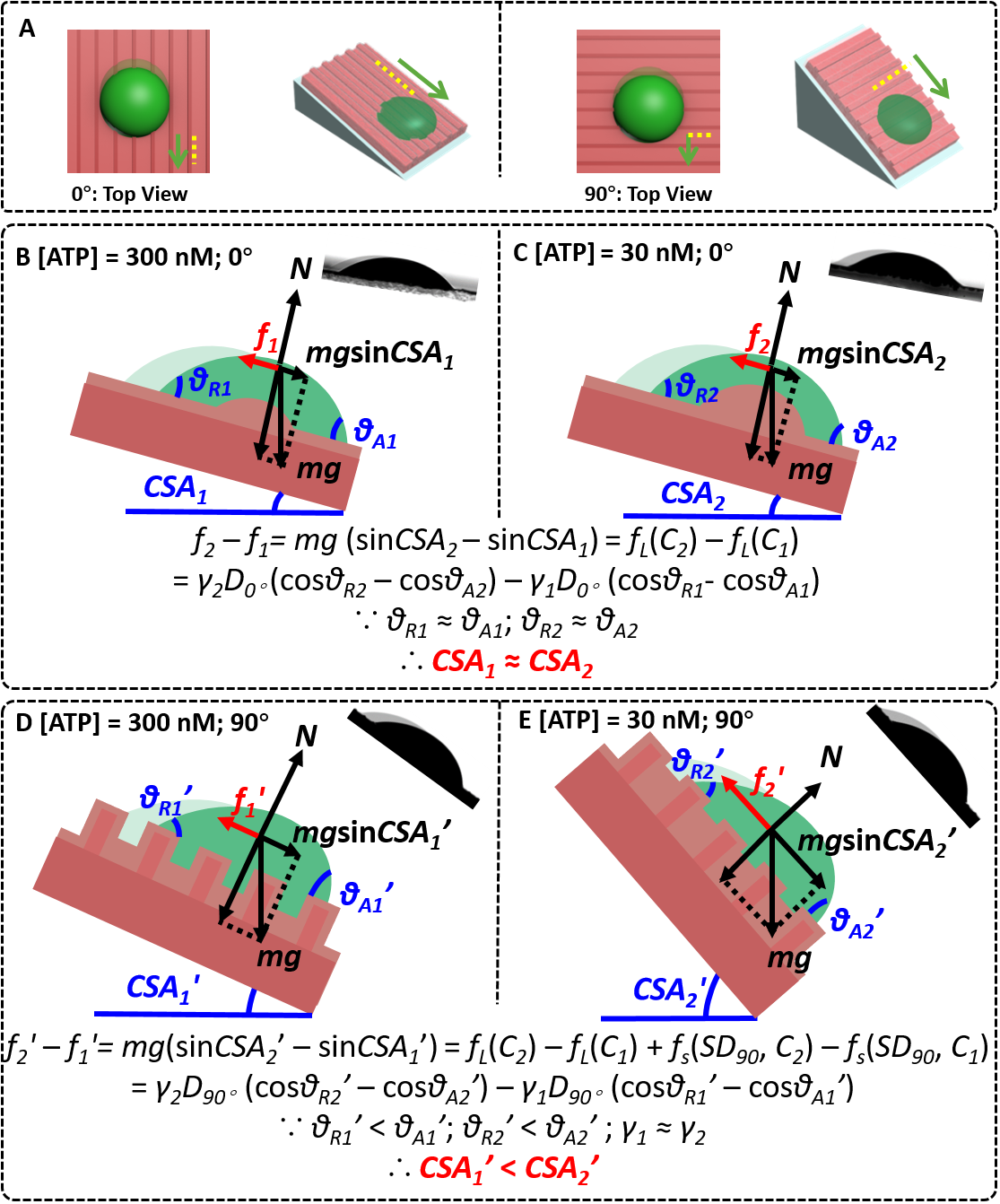
**Figure S12.** Force Analyzing of RCA Droplets with Small Difference in ATP Concentration in 0° and 90° Sliding Directions. (A) Schematic diagram of RCA droplets in 0° and 90° sliding directions. (B-E) Force analyzing illustration of RCA droplets. The droplets with small difference in ATP concentrations sitting on tilted periodic micro-grooves structural slippery surface in 0° direction (B, C) and 90° directions (D, E), respectively.


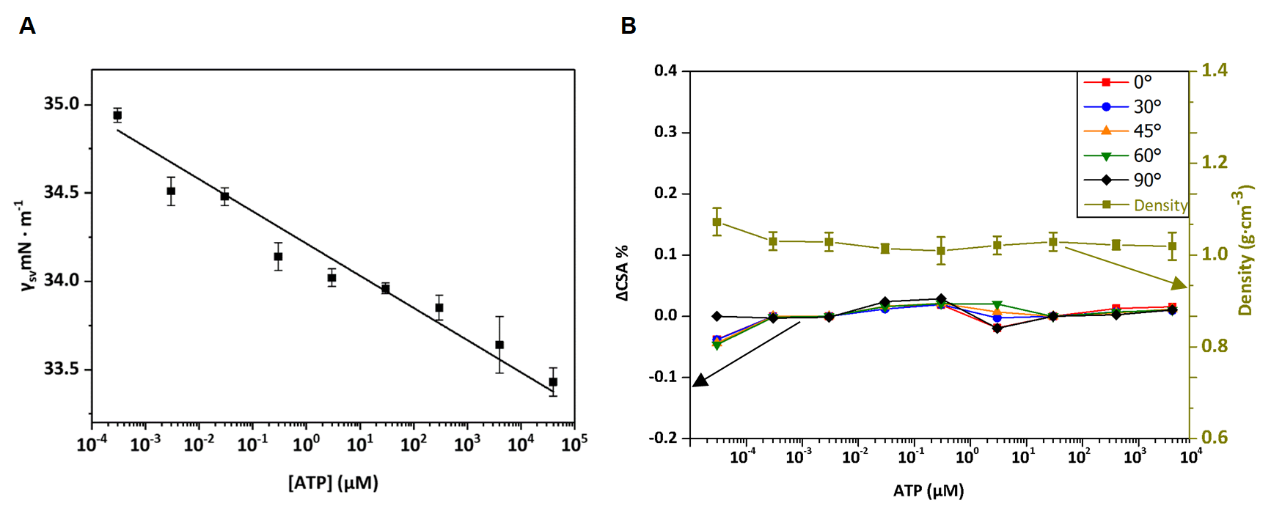
**Figure S13.** (A) Relationship between surface tension and ATP concentration of RCA droplet, with adjusted R-square of 0.96107. (B) Difference in CSA and droplet’s density as functions of ATP concentration, in 5 different sliding directions. The average density was adopted for the dependence ATP concentration. Where$, \Delta CSA\%=\frac{\mathrm{CSA} -{CSA}_{average}}{CSA}$ .


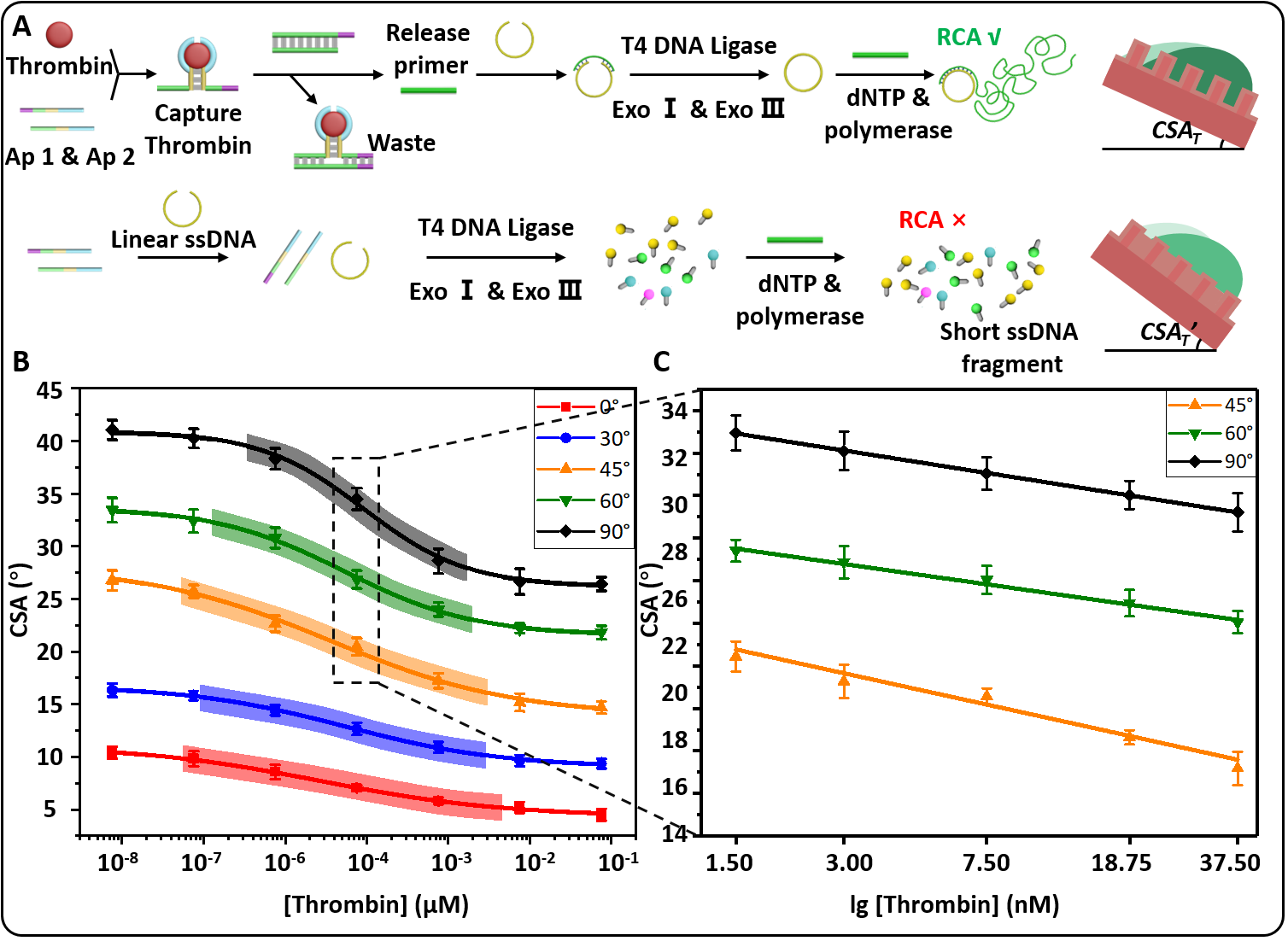
**Figure S14.** Thrombin detection was realized by the Thrombin-triggered RCA reaction. Thrombin-binding aptamers and coupled DNA strand-displacement cascades were designed. In our experiment, the CSAs of RCA droplets triggered by Thrombin with different concentrations were tested. Various detection dynamic ranges were also realized in five sliding directions (**Table S2**), and the widest detection dynamic range was 8500-fold (54.2 pM to 459 nM) in 0° sliding direction. With the steeper slope of normalized CSA curves response to Thrombin’s concentration in sliding directions of 30°, 45°, and 90°, RCA droplets with 2-fold change in Thrombin concentrations (between 1.50 nM and 3.00 nM) can be distinguished. (A) Working principle of detecting Thrombin based on RCA droplet’s motion behaviors.(B) CSAs for RCA droplets with various Thrombin concentrations (7.5 pM, 75 pM, 750 pM, 7.5 nM, 75 nM, 750 nM and 7.5 μM) sliding in five different directions, respectively. The colorful shadows are the dynamic ranges of detection Thrombin in different sliding directions. (C) In sliding directions of 45°, 60°and 90°, RCA droplets with different concentrations of Thrombin (1.50 nM, 3.00 nM, 7.50 nM, 18.75 nM and 37.50 nM) concentrations can be sensitively detected, and droplets with 2-fold change in Thrombin concentrations can be distinguished.


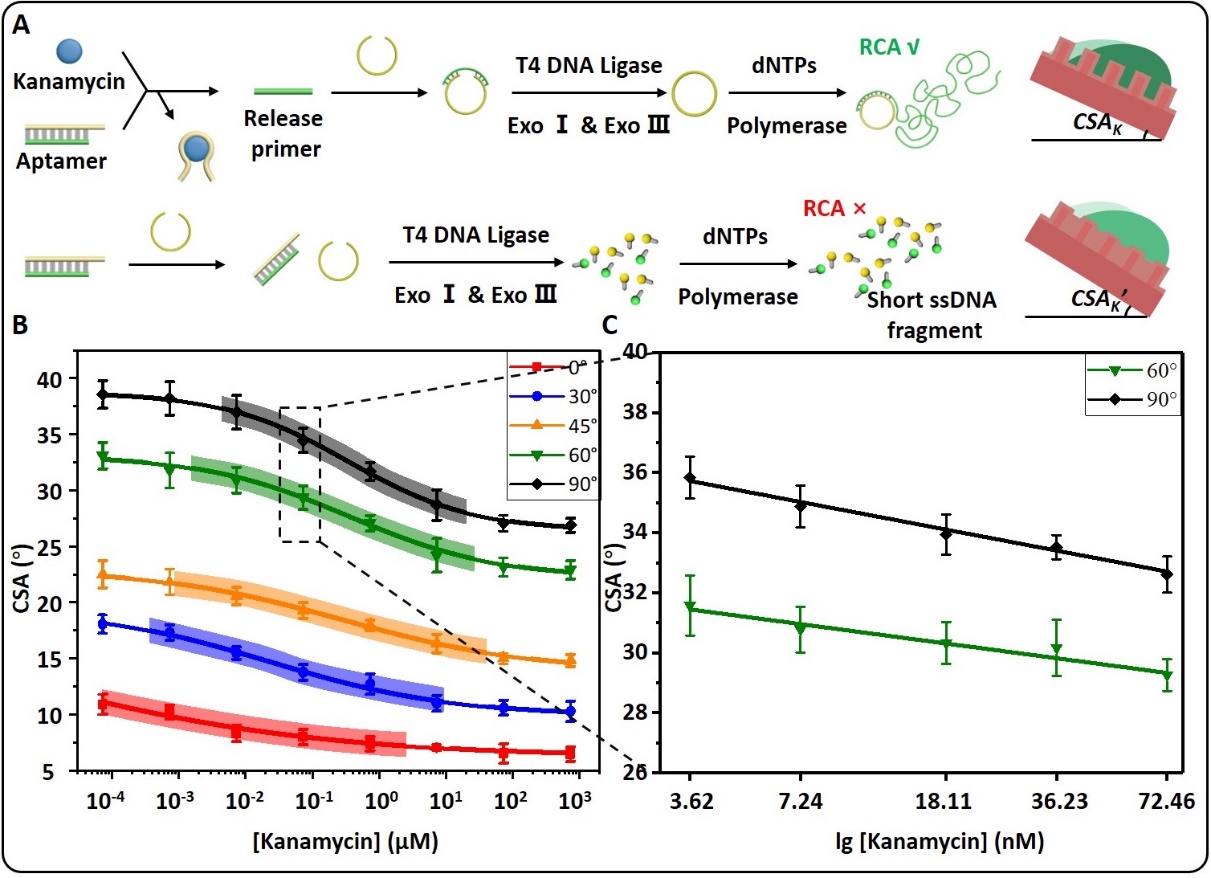
**Figure S15.** Kanamycin detection was also realized. In this experiment, 26 bases DNAzyme was designed as Kanamycin recognition aptamer, 13 bases DNAzyme was designed as primer, and 83 bases single linear ssDNA was modified with phosphorylation as RCA template. Specifically, the aptamer and the primer were well matched in the form of Aptamer/Primer double stranded DNA. A series of RCA droplets with various Kanamycin concentrations were prepared, including 72.4 pM, 724 pM, 7.24 nM, 72.4 nM, 724 nM, 7.24 μM, 72.4 μM and 724 μM. Then, their CSAs in five different sliding directions on the periodic micro-grooves slippery surface were tested. The dynamic ranges of detection were shown in **Table S2**. In the sliding direction of 0°, the widest dynamic range, 34200-fold with Kanamycin concentrations between 72.4 pM and 2.47 μM, was achieved. To demonstrate the sensitive detection in 60° and 90° sliding directions, RCA droplets with Kanamycin concentration of 3.62 nM, 7.24 nM, 18.11 nM, 36.23 nM, 72.46 nM were tested. The result showed RCA droplets with 2-fold change in Kanamycin’s concentration (near 7.24 nM) can be distinguished. Taking together, the sensitive detection of miRNAs, Thrombin, and Kanamycin demonstrate the dual-regulation strategy is universal to major analytes classes. (A) Working principle of detecting Kanamycin. (B) CSAs for various Kanamycin concentrations (72.4 pM, 724 pM, 7.24 nM, 72.4 nM, 724 nM, 7.24 μM, 72.4 μM and 724μM) in five different sliding directions, respectively. The colorful shadows are the dynamic ranges of detection Kanamycin in different sliding directions. (C) In sliding directions of 60° and 90°, RCA droplet with different concentrations of Kanamycin (3.62 nM, 7.24 nM, 18.11 nM, 36.23 nM and 72.46 nM) can be sensitively detected, and droplets with 2-fold change in Kanamycin concentrations (near 7.24 nM) can be distinguished.

**Table S1.** Adopted DNA sequences.

| Oligonucleotide name | Sequence (5’ to 3’) description |
| --- | --- |
| Linear ssDNA (ATP) | *Phosphate-*TCGTTTGATGTTCCTAACGTACCAACGCACACGCA  GTATTATGGACTGGTAAAAGCTTTCCGAGGTAGCCTGGAGCATAGAGGCATTGGCTG |
| Primer (for ATP) | TAGGAACATCAAACGACAGCCA |
| Linear ssDNA  (miR-21) | *Phosphate-*TCAGTCTGATAAGCTAACGTACCAACGCACACGC  AGTATTATGGACTGGTAAAAGCTTTCCGAGGTAGCCTGGAGCATAGAGGCATTCAACA |
| Aptamer (miR-21) | UAGCUUAUCAGACUGAUGUUGA |
| SM | UAGCUUAUCAGACUGAUGUAGA |
| DM | UAGCUUAUAAGACUGAUGUAGA |
| TM | UAGCAUAUCAGGCUGAUGUAGA |
| RM | AGCUACAUUGUCUGCUGGGUUC |
| Linear ssDNA  (Thrombin) | *Phosphate-*TCGTTTGATGTTCCTAACGTACCAACGCACACGCA  GTATTATGGACTGGTAAAAGCTTTCCGAGGTAGCCTGGAGCATAGAGGCATTGGCTG |
| Primer (Thrombin) | TAGGAACATCAAACGACAGCCA |
| PC probes | AAAAAAAATGGCTGTCGTTTGATGTTCCTA |
| Ap-1 | TAGGAACATCAAACGTTGATTCGGTTGGT |
| Ap-2 | GTGGTTGGGAATCAAACAGCCATTTTTTTT |
| Linear ssDNA  (Kanamycin) | *Phosphate-*GAGTCCCACGTACCAACGCACACGCAGTATTATG  GACTGGTAAAAGCTTTCCGAGGTAGCCTGGAGCATAGAGGCATGGTAAT |
| Primer (Kanamycin) | GGGACTCATTACC |
| Aptamer (Kanamycin) | GGGACTTGGTTTAGGTAATGAGTCCC |

**Table S2.** Detection dynamic ranges and sensitivity of ATP, miRNA, Kanamycin, and Thrombin on micro-grooves structural surfaces with various sliding directions.

| Detection | Sliding Direction | Dynamic Range  (μM) | Fold | Sensitivity (Slope) | R^2^ of linear  fitting |
| --- | --- | --- | --- | --- | --- |
| ATP | 0° | 5.26 × 10^-4^ - 5.19 × 10^2^ | 9.87 × 10^5^ | -0.80 | 0.994 |
|  | 30° | 1.01 × 10^-3^ - 3.64 × 10^2^ | 3.59 × 10^5^ | -1.01 | 0.994 |
|  | 45° | 6.11 × 10^-4^ - 2.11 × 10^2^ | 3.46 × 10^5^ | -1.36 | 0.994 |
|  | 60° | 9.76 × 10^-4^ - 7.98 × 10^1^ | 8.17 × 10^4^ | -1.76 | 0.993 |
|  | 90° | 6.46 × 10^-4^ - 1.88 × 10^0^ | 2.90 × 10^3^ | -3.14 | 0.992 |
| miR-21 | 0° | 9.26 × 10^-6^ - 4.42 × 10^-1^ | 4.77 × 10^4^ | -0.97 | 0.999 |
|  | 30° | 8.67 × 10^-5^ - 2.91 × 10^-1^ | 7.20 × 10^3^ | -1.49 | 0.995 |
|  | 45° | 5.20 × 10^-5^ - 3.03 × 10^-1^ | 8.10 × 10^3^ | -2.11 | 0.993 |
|  | 60° | 1.23 × 10^-4^ - 1.95 × 10^-1^ | 2.42 × 10^4^ | -2.11 | 0.998 |
|  | 90° | 3.23 × 10^-4^ - 1.70 × 10^-1^ | 2.80 × 10^3^ | -3.39 | 0.996 |
| Thrombin | 0° | 5.42 × 10^-5^ - 4.59 × 10^-1^ | 8.50 × 10^3^ | -1.29 | 0.995 |
|  | 30° | 8.67 × 10^-5^ - 2.91 × 10^-1^ | 3.40 × 10^3^ | -1.72 | 0.996 |
|  | 45° | 5.20 × 10^-5^ - 3.03 × 10^-1^ | 5.80 × 10^3^ | -2.79 | 0.997 |
|  | 60° | 1.23 × 10^-4^ - 1.95 × 10^-1^ | 1.60 × 10^3^ | -3.22 | 0.995 |
|  | 90° | 3.23 × 10^-4^ - 1.69 × 10^-1^ | 5.00 × 10^2^ | -4.79 | 0.993 |
| Kanamycin | 0° | 7.24 × 10^-5^ - 2.47 × 10^0^ | 3.24 ×10^4^ | -0.86 | 0.977 |
|  | 30° | 3.52 × 10^-4^ - 9.29 × 10^0^ | 2.63 × 10^4^ | -1.54 | 0.995 |
|  | 45° | 8.41 × 10^-4^ - 3.97 × 10^1^ | 4.71 × 10^4^ | -1.43 | 0.997 |
|  | 60° | 1.48 × 10^-3^ - 2.65 × 10^1^ | 1.79 × 10^4^ | -2.10 | 0.997 |
|  | 90° | 4.22 × 10^-3^ - 1.98 × 10^1^ | 4.70 × 10^4^ | -2.81 | 0.995 |

**Table S3.** The jack-knifed classification procedure based on dual-regulation stragety.

|  | Lamda DNA | Linaear  DNA | | Fish Sperm  DNA | M13 | PUC  119 | | Control | Correct |
| --- | --- | --- | --- | --- | --- | --- | --- | --- | --- |
| Lamda DNA | 8 | 0 | | 0 | 0 | 0 | | 0 | 100% |
| Linear  DNA | 0 | 8 | | 0 | 0 | 0 | | 0 | 100% |
| Fish Sperm  DNA | 0 | 0 | | 8 | 0 | 0 | | 0 | 100% |
| M13 | 0 | 0 | | 0 | 8 | 0 | | 0 | 100% |
| PUC  119 | 0 | 0 | | 0 | 0 | 8 | | 0 | 100% |
| Control | 0 | 0 | | 0 | 0 | 0 | | 8 | 100% |
| Total | 8 | 8 | | 8 | 8 | 8 | | 8 | 100% |
| N = 48 | | | N Correct = 48 | | | | Proportion Correct  = 1.000 | | |

Summary of 48 samples classification to 5 DNAs and 1 control analytes with cross-validation by LDA.

**Table S4.** The relationships between CAs and ATP concentrations (300 pM, 3 nM, 30 nM, 300 nM, 3 μM, 30 μM, 300 μM, 4 mM and 40 mM) of RCA droplets on periodic micro-groove slippery surface with sliding directions of 0°, 30°, 45°, 60°, and 90°, respectively.

| Sliding Direction  CA (°) | 0° | 30° | 45° | 60° | 90° |
| --- | --- | --- | --- | --- | --- |
|  |  |  |  |  |  |
|  |  |  |  |  |  |
| [ATP] = 40 mM | 45.5 ± 0.6 | 50.7 ± 1.5 | 50.2 ± 0.5 | 49.6 ± 1.3 | 50.5 ± 1.5 |
| [ATP] = 4 mM | 47.1 ± 0.2 | 50.7 ± 1.2 | 50.9 ± 1.0 | 50.3 ± 1.4 | 49.5 ± 1.2 |
| [ATP] = 300 μM | 48.2 ± 1.5 | 51.3 ± 1.0 | 50.7 ± 1.1 | 50.5 ± 1.5 | 50.9 ± 1.5 |
| [ATP] = 30 μM | 48.7 ± 1.1 | 51.0 ± 0.9 | 51.0 ± 1.7 | 51.0 ± 1.1 | 51.7 ± 1.4 |
| [ATP] = 3 μM | 46.2 ± 1.4 | 51.4 ± 1.2 | 53.0 ± 1.3 | 51.2 ± 1.5 | 51.6 ± 0.7 |
| [ATP] = 300 nM | 47.7 ± 1.1 | 50.8 ± 1.1 | 53.3 ± 1.3 | 51.0 ± 1.5 | 52.1 ± 1.3 |
| [ATP] = 30 nM | 46.4 ± 1.3 | 52.1 ± 1.2 | 53.0 ± 1.7 | 52.5 ± 0.9 | 51.7 ± 1.6 |
| [ATP] = 3 nM | 48.0 ± 1.1 | 52.0 ± 1.3 | 55.7 ± 1.7 | 52.0 ± 0.8 | 53.2 ± 1.5 |
| [ATP] = 300 pM | 47.0 ± 0.9 | 48.0 ± 1.4 | 49.5 ± 0.4 | 49.6 ± 1.2 | 49.7 ± 1.7 |

**Table S5.** Total resistance of RCA droplets with various ATP concentrations slide in five different directions based on experimental CSAs.

| *f* (μN) | 0° | 30° | 45° | 60° | 90° |
| --- | --- | --- | --- | --- | --- |
| [ATP] = 40 mM | 2.4 | 4.4 | 6.2 | 7.2 | 11.1 |
| [ATP] = 4 mM | 2.5 | 4.4 | 6.2 | 7.2 | 11.2 |
| [ATP] = 300 μM | 2.7 | 4.7 | 6.6 | 7.6 | 11.3 |
| [ATP] = 30 μM | 3.0 | 4.9 | 6.7 | 7.6 | 11.3 |
| [ATP] = 3 μM | 3.1 | 5.2 | 7.2 | 8.0 | 11.5 |
| [ATP] = 300 nM | 3.3 | 5.4 | 7.5 | 8.7 | 11.9 |
| [ATP] = 30 nM | 3.6 | 5.9 | 8.0 | 9.4 | 12.9 |
| [ATP] = 3 nM | 4.2 | 6.4 | 8.7 | 9.8 | 13.9 |
| [ATP] = 300 pM | 4.5 | 6.8 | 9.4 | 10.7 | 15.1 |

For a droplet to move on a surface tilted at an angle *α*, the tangential component of the gravitational force, mgsin*α*, acting on the droplet has to exceed the resistance, *f*. In our experiment, *α* equals to CSA, and *f* is the total resistance coming from liquid phase (liquid resistance) and solid phase (solid resistance). *f* can be calculated by experimental CSAs.

**Table S6.** Additional solid resistance compared to RCA droplet sliding in direction of 0°. Various additional solid resistance of RCA droplets sliding in direction of 30°, 45°, 60°, and 90°compared to in 0°, which comes from anisotropic surface resistance.

| Solid Resistance ($f_{s}$) (μN) | 0° | 30° | 45° | 60° | 90° |
| --- | --- | --- | --- | --- | --- |
| [ATP] = 40 mM | Ground  State | 2.0 | 3.8 | 4.8 | 8.7 |
| [ATP] = 4 mM | N/A | 1.9 | 3.8 | 4.8 | 8.7 |
| [ATP] = 300 μM | N/A | 2.0 | 3.9 | 4.9 | 8.6 |
| [ATP] = 30 μM | N/A | 1.9 | 3.7 | 4.6 | 8.3 |
| [ATP] = 3 μM | N/A | 2.0 | 4.1 | 4.8 | 8.4 |
| [ATP] = 300 nM | N/A | 2.2 | 4.3 | 5.4 | 8.7 |
| [ATP] = 30 nM | N/A | 2.3 | 4.4 | 5.8 | 9.3 |
| [ATP] = 3 nM | N/A | 2.2 | 4.5 | 5.6 | 9.7 |
| [ATP] = 300 pM | N/A | 2.3 | 4.9 | 6.3 | 10.6 |
| Average additional  solid resistance ($\bar{f_{S}}$) | N/A | 2.1 ± 0.2 | 4.0 ± 0.4 | 5.2 ± 0.5 | 9.0 ± 0.8 |

In this experiment, when RCA droplets slide in direction of 0° with ATP concentration of 40 mM, the RCA droplet was suffered the lowest solid resistance and the lowest liquid resistance. Considering this case as a ground state, for RCA droplets slide in other directions or/and contain lower centration of ATP, they suffered additional resistance from solid surface or/and liquid phase. Additional solid resistances of RCA droplets with the same ATP concentration were can be calculated by compared the resistance between 0° and other sliding directions in Table S4. As a result, average additional solid resistance can be obtained: 2.1 ± 0.2 μN, 4.0 ± 0.4 μN, 5.2 ± 0.5 μN, 9.0 ± 0.8 μN for direction of 30°, 45°, 60°, and 90°, respective.

**Table S7**. Additional liquid resistance compared to RCA droplet with 40 mM ATP. The additional liquid resistance mainly comes from hydrophobic interaction.

| Liquid Resistance ($f_{L}$) (μN) | 0° | 30° | 45° | 60° | 90° | Average Additional  Liquid Resistance ($\bar{\boldsymbol{f}_{\boldsymbol{l}}}$) |
| --- | --- | --- | --- | --- | --- | --- |
| [ATP] = 40 mM | Ground State | N/A | N/A | N/A | N/A | N/A |
| [ATP] = 4 mM | 0.0 | 0.0 | 0.0 | 0.1 | 0.1 | 0.0 ± 0.1 |
| [ATP] = 300 μM | 0.3 | 0.4 | 0.4 | 0.4 | 0.1 | 0.3 ± 0.1 |
| [ATP] = 30 μM | 0.6 | 0.5 | 0.5 | 0.4 | 0.1 | 0.4 ± 0.2 |
| [ATP] = 3 μM | 0.7 | 0.8 | 1.0 | 0.7 | 0.4 | 0.7 ± 0.2 |
| [ATP] = 300 nM | 0.8 | 1.1 | 1.3 | 1.5 | 0.7 | 1.1 ± 0.3 |
| [ATP] = 30 nM | 1.2 | 1.5 | 1.8 | 2.2 | 1.7 | 1.7 ± 0.3 |
| [ATP] = 3 nM | 1.8 | 2.0 | 2.5 | 2.6 | 2.7 | 2.3 ± 0.4 |
| [ATP] = 300 pM | 2.1 | 2.5 | 3.2 | 3.5 | 3.9 | 3.0 ± 0.7 |

Additional liquid resistances of RCA droplets with the different ATP concentration were calculated by compared the resistance between ATP concentration of 40 mM and other concentration in Table S4. As a result, average additional liquid resistance can be obtained: 0.0 ± 0.1 μN, 0.3 ± 0.1 μN, 0.4 ± 0.2 μN, 0.7 ± 0.2 μN, 1.1 ± 0.3 μN, 1.7 ± 0.3 μN, 2.3 ± 0.4 μN, and 3.0 ± 0.7 μN for ATP concentration of 4 mM, 300 μM, 30 μM, 3 μM, 300 nM, 30 nM, 3 nM, and 300 pM, respectively (**Table R4**).

**Table S8.** The calculated CSAs based on additional solid and liquid resistance.

| *θ*c | 0° | 30° | 45° | 60° | 90° |
| --- | --- | --- | --- | --- | --- |
| [ATP] = 40 mM | 7.0 | 13.0 | 19.1 | 22.4 | 34.6 |
| [ATP] = 4 mM | 7.1 | 13.1 | 19.2 | 22.5 | 34.7 |
| [ATP] = 300 μM | 7.9 | 13.9 | 20.0 | 23.3 | 35.7 |
| [ATP] = 30 μM | 8.2 | 14.3 | 20.4 | 23.7 | 36.1 |
| [ATP] = 3 μM | 9.0 | 15.1 | 21.2 | 24.5 | 37.1 |
| [ATP] = 300 nM | 10.1 | 16.2 | 22.4 | 25.7 | 38.4 |
| [ATP] = 30 nM | 11.8 | 17.9 | 24.2 | 27.6 | 40.6 |
| [ATP] = 3 nM | 13.7 | 19.9 | 26.3 | 29.7 | 43.1 |
| [ATP] = 300 pM | 15.8 | 22.0 | 28.5 | 32.1 | 45.9 |

**Table S9.** The difference between experimental and calculated CSAs based on additional solid and liquid resistance.

| Deviation Percentage (%) | 0° | 30° | 45° | 60° | 90° |
| --- | --- | --- | --- | --- | --- |
| [ATP] = 40 mM | Ground State | -3.0 | -5.3 | -5.7 | -2.0 |
| [ATP] = 4 mM | -0.1 | -3.8 | -5.8 | -5.7 | -2.1 |
| [ATP] = 300 μM | -2.4 | -2.9 | -4.7 | -5.3 | -4.7 |
| [ATP] = 30 μM | 4.4 | -1.1 | -4.0 | -5.5 | -5.0 |
| [ATP] = 3 μM | 0.9 | 0.3 | 0.3 | -4.3 | -4.2 |
| [ATP] = 300 nM | -7.0 | -2.3 | -0.9 | 0.6 | -4.5 |
| [ATP] = 30 nM | -12.9 | -5.8 | -3.8 | 0.3 | -2.3 |
| [ATP] = 3 nM | -12.6 | -7.5 | -3.0 | -2.2 | 0.7 |
| [ATP] = 300 pM | -22.1 | -14.2 | -7.1 | -4.3 | -0.2 |

**Table S10**. CA hysteresis (Δ*θ*) of RCA droplets sliding on the micro-grooves structural slippery surface with different ATP concentrations and various sliding directions, where Δ*θ = θ_R_ – θ_A_.* Except 90° sliding direction, Δ*θ* were less than 10°, according with Dussan’s assumption.

| *Δθ*  [ATP] | 0° | 30° | 45° | 60° | 90° |
| --- | --- | --- | --- | --- | --- |
| [ATP] = 40 mM | 1.7 ± 0.5° | 3.2 ± 0.3° | 4.9 ± 0.3° | 7.1 ± 0.4° | 11.0 ± 0.3° |
| [ATP] = 4 mM | 2.8 ± 0.2° | 3.7 ± 0.3° | 5.3 ± 0.4° | 7.6 ± 0.5° | 11.9 ± 0.2° |
| [ATP] = 300 μM | 2.7 ± 0.1° | 4.0 ± 0.4° | 5.6 ± 0.4° | 7.8 ± 0.5° | 12.5 ± 0.3° |
| [ATP] = 30 μM | 2.7 ± 0.3° | 4.3 ± 0.5° | 6.6 ± 0.5° | 7.8 ± 0.2° | 13.8 ± 0.5° |
| [ATP] = 3 μM | 3.3 ± 0.4° | 4.6 ± 0.2° | 6.7 ± 0.3° | 8.0 ± 0.4° | 13.8 ± 0.4° |
| [ATP] = 300 nM | 3.7 ± 0.3° | 4.7 ± 0.4° | 6.8 ± 0.3° | 8.3 ± 0.2° | 14.7 ± 0.3° |
| [ATP] = 30 nM | 3.5 ± 0.5° | 5.3 ± 0.5° | 7.3 ± 0.6° | 8.5 ± 0.5° | 14.9 ± 0.4° |
| [ATP] = 3 nM | 4.5 ± 0.3° | 5.4 ± 0.3° | 8.0 ± 0.3° | 8.6 ± 0.3° | 15.2 ± 0.2° |
| [ATP] = 300 pM | 5.5 ± 0.2° | 6.3 ± 0.5° | 9.0 ± 0.2° | 9.0 ± 0.2° | 20.4 ± 0.4° |

**Table S11.** Receding angle (*θ_R_*), advancing angle (*θ_A_*)*_,_* CA Hysteresis, and CSAs of RCA droplets with ATP concentrations of 300 nM (*C*_1_) and 30 nM (C_2_) when sliding in 0° or 90° directions, respectively.

|  | 0° Sliding Direction | | | |
| --- | --- | --- | --- | --- |
|  | *θ_R_* | *θ_A_* | CA Hysteresis | *CSA* |
| C_1_ = 300 nM | 45.2 ± 0.7° | 48.9 ± 0.1° | 3.7 ± 0.3° | 9.4 ± 0.4° |
| C_2_ = 30 nM | 44.2 ± 0.3° | 47.7 ± 0.2° | 3.5 ± 0.5° | 10.3 ± 0.6° |
|  | 90° Sliding Direction | | | |
|  | *θ_R’_* | *θ_A’_* | CA Hysteresis | *CSA’* |
| C_1_ = 300 nM | 42.2 ± 0.6° | 56.9 ± 0.8° | 14.7 ± 0.3° | 36.7 ± 1.3° |
| C_2_ = 30 nM | 44.5 ± 0.9° | 59.4 ± 1.0° | 14.9 ± 0.4° | 39.7 ± 2.0° |

**Materials**

All the Linear ssDNAs, primers and aptamers used in this work, including ATP, AMP, GTP, UTP and CTP were provided by Takara Biomedical Technology (Beijing) Co., Ltd. T4 DNA ligase, exonuclease I (Exo I), exonuclease III (Exo III), phi 29 DNA polymerase (phi-29 DNA), deoxynucleotide solution mixture (dNTPs) were obtained from Thermo Fisher Scientific Inc. (Waltham, MA, USA). Lambda DNA, M13 and pCU19 were bought from NEB. Fish sperm DNA was bought from Sigma Aldrich. (St. Louis, MO, USA). Polydimethylsiloxane (PDMS) oligomer and cross-linker (Sylgard 184) were purchased from Dow Corning Corp. (Midland, Mi, USA). 1H,1H,2H,2H-Perfluorooctyltrichlorosilane was bought from Macklin Biochemical Co. Ltd (FAS, Shanghai, China). N-decane was bought from Aladdin Industrial Corporation (Shanghai, China). The DNA sequences were listed in Table S1.

Preparation of Micro-Grooves Structural PDMS Slippery Surface

The micro-grooves structural PDMS substrate was prepared by the silicon replica-molding process (Figure S1). The periodically micro-grooves silicon wafer template was designed with 20 μm width, 20 μm height and six different spacing specifications, including 10 μm, 50 μm, 100 μm, 200 μm, 300μm, 400 μm were prepared with chrome plate mask photolithographic method. The adopted lubricant was n-decane. Taking substrate with 10 μm of width, 20 μm of height, 200 μm of spacing for example, the obtained micro-grooves structured silicon template was modified with 80 μL 1H,1H,2H,2H-Perfluorooctyltrichlorosilane, treated in an evacuated desiccator at 80°C for 3h. Then, the PDMS pre-polymer and cross linker were mixed homogeneously (with weight ratio of 10:1) and degassed for 15 min under vacuum. The obtained mixture was poured over the FAS modified structured silicon template and adopted spin-coating method at 500 r/min for 30 seconds, and then heated at 80°C for 60 mins in a vacuum. After that, the PDMS substrate was achieved by carefully peeling off from the silicon substrate. The as-prepared PDMS substrate was cut into 2 cm × 3 cm, and then immersed into n-decane at room temperature for 6 h.

The Preparation of RCA Droplet Detection of ATP.

Firstly, phosphorylated linear ssDNA (1 μL, 30 μM) and 3 μL of 30 μM primer probes were mixed in 36 μL hybridization buffer (pH 7.4, 10 mM Tris-HCl containing 100 mM NaCl, 5 mM MgCl_2_). The mixture was heated to 95°C for 10 min and then cooled down to room temperature slowly to make sureDNA fold properly. Secondly, adding 10 μL T4 DNA ligase with concentration of 0.5 U/μL of and 10 μl T4 DNA ligase buffer to the resultant solution. The ligation was incubated in a total volume of 96 μL at 22°C for 2 h. The T4 DNA ligase was made of pH 7.6, 66 mM Tris-HCl, 0.6 mM MgCl_2_, 3.3 μM Na4P_2_O_7_, 10 mM DTT. Thirdly, add 40 U of Exo I and Exo III to the ligation solution, incubated under 37°C for 3 h. Then, incubating the sample at 80°C for 20 min to inactivate the T4 DNA ligase and exonucleases. Finally, adding 25 mM dNTP mixture to the above reaction and incubated at 37°C for 2 h. The adopted RCA procedure time is 2 h due to the obvious difference of SA before and after the RCA process according to our previous research.^4^ Meanwhile, reaction time in every step was well controlled in every RCA processes to avoid variation in CSAs caused by other parameters instead of ATP’s concentration. The resulting solution was heated to 65°C for 20 min to inactivate the DNA polymerase before measurement. The ATP concentrations were 40 mM, 4 mM, 300 μM, 30 μM, 3 μM, 300 nM, 30 nM, 3 nM, 300 pM and 0. For the ATP detection in serum sample, 10 μL diluted serum was added at the initial stage.

Detection of miR-21.

The miR-21 RCA droplets were prepared similar to ATP RCA droplet, by adopting the sequence of miR-21 and linear ssDNA (miR-21) instead of primer and linear ssDNA respectively, with various miRNA concentrations and aseptic conditions. The miR-21 concentrations were 2.08 μM, 208 nM, 20.8 nM, 2.08 nM, 208 pM, 20.8 pM and 2.08 pM, respectively.

Detection of Thrombin.

10 μL of 20 μM primer and primer complement probes (PC) were well mixed and incubated at 95°C for 5 min, respectively. Then, the solution slowly cooled to room temperature to prepare PC/primer double-stranded DNA. 10 μM aptamer (Ap-1 and Ap-2) were incubated with different concentrations of thrombin at 37°C for 1 h. The PC/primer solution was then added and incubated for another 1 h. After that, the RCA reaction was conducted follow the above-mentioned details, but with the modification of Thrombin concentration. The concentrations of Thrombin were 7.5 μM, 750 μM, 75 μM, 7.5 μM, 750 nM, 75 nM, 7.5 nM, respectively.

Detection of Kanamycin.

The process of Kanamycin RCA droplets was carried out with an aptamer, a DNA primer and a single linear phosphorylated DNA. Firstly, the aptamer and the short DNA primer combined with each other to form double strand probe. Then, the primer released with the addition of the target of kanamycin, and circle the phosphorylated ssDNA under the help of T4 ligation. After that, the mixtures made the DNA amplification under isothermal environment using phi-29 DNA polymerase through strong strand-displacement. The concentration of kanamycin included 724 μM, 72.4 μM, 7.24 μM, 724 nM, 72.4 nM, 7.24 nM, 724 pM and 72.4 pM.

Preparation of Cell Culture and Cell Lysates

PC-3 cells were cultured with typically method, 90% 1640 (GIBCO) medium with 10% fetal calf serum and 1% penicillin streptomycin (PS, 10000 IU penicillin and 10000 μg/mL streptomycin, MULTICELL) in a humidified atmosphere containing 5% CO_2_ at 37°C. PC-3 cells were seeded on glass-bottom culture dishes (Nest, China) for 6h, 36h, 48h and 70h to reach densities of 8.0×10^4^ cells/mL, 7.0 ×10^5^ cells/mL, 9.0×10^5^ cells/mL and 5.0×10^6^ cells/mL. Then, PC-3 cells were lysed with more than five successive cycles quenching heating steps, frozen in liquid nitrogen followed by thawing in a 60°C water bath. The ATP lysate was the upper phase after centrifuging the above PC-3 solutions at RCF 10,000 x g for 3 min.

**Definition of Dynamic Range and Sensitivity**


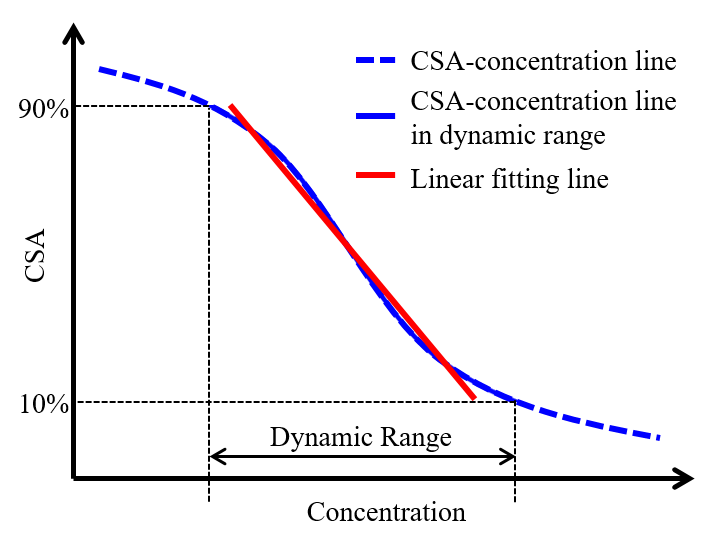


Dynamic range is defined as the range of target’s concentration with relative CSA occupancy between 10% and 90%. Sensitivity is defined as change in response per unit change in target concentration’s dynamic range, which also can be presented as the slope of linear fitting line (apparent fit, the red line in figure) of dynamic range. Specific data were listed in Table S2.

Proof of Orthogonality between Additional Solid Resistance and Additional Liquid Resistance

We export table S4 as a matrix in the same order,

$$F_{c,d}=\left[ \begin{matrix} F_{c1,d1} & F_{c1,d2} & \ldots& F_{c1,d5} \\ F_{c2,d1} & \ldots& \ldots& \ldots\\ \ldots& \ldots& \ldots& \ldots\\ F_{c9,d1} & \ldots& \ldots& F_{c9,d5} \end{matrix} \right]$$

c means ATP’s concentration and d means sliding direction in the matrix.

Firstly, in the first col of the matrix $F_{c,d}$, subtract first element ($F_{c1,d1}$) in the first col from every element ($F_{c2,d1}, F_{c3,d1}\ldots F_{c9,d1}$) , and we have

$\left\{ \begin{aligned} F_{c2,d1}-F_{c1,d1}={\Delta F}_{\Delta c1,d1} \\ F_{c3,d1}-F_{c1,d1}={\Delta F}_{\Delta c2,d1} \\ \ldots\\ F_{c9,d1}-F_{c1,d1}={\Delta F}_{\Delta c8,d1} \end{aligned} \right.$.

Within the allowable error range, there is ${\Delta F}_{\Delta c1, d1}$=${\Delta F}_{\Delta c2, d1}$=$\ldots\text{=}{\Delta F}_{\Delta c7, d1}$=${\Delta F}_{\Delta c8, d1}$(Table S5), and from $\Delta c1\neq$ $\Delta c$2 $\neq\ldots\neq\Delta c$7 $\neq$ $\Delta c$8, we have ${\Delta F}_{\Delta c1,d1}$ independent from $\Delta c$. With the same argument, we have ${\Delta F}_{\Delta c,d2}$, ${\Delta F}_{c3,\Delta d}$…${\Delta F}_{c9,\Delta d}$ are related to d and independent from $\Delta c$. (${\Delta F}_{\Delta c,d}$=${\Delta f}_{S}(d)$)

Secondly, in the first row, subtract first element ($F_{c1,d1}$) in the first row from every element ($F_{c1,d2}$, $F_{c1,d3}$…$F_{c1,d5}$), and we have

$\left\{ \begin{aligned} F_{c1,d2}-F_{c1,d1}={\Delta F}_{c1,\Delta d1} \\ F_{c1,d3}-F_{c1,d1}={\Delta F}_{c1,\Delta d2} \\ \ldots\\ F_{c1,d4}-F_{c1,d1}={\Delta F}_{c1,\Delta d4} \end{aligned} \right.$,

Within the allowable error range, threre is ${\Delta F}_{c1,\Delta d1}$=${\Delta F}_{c1,\Delta d2}$=${\Delta F}_{c1,\Delta d3}$=${\Delta F}_{c1,\Delta d4}$(Table S6), and from $\Delta d1\neq$ $\Delta d$2 $\neq$ $\Delta d$3 $\neq$ $\Delta d$4, we have ${\Delta F}_{c1,\Delta d}$ is independent from $\Delta d$. With the same argument, we have ${\Delta F}_{c2,\Delta d}$, ${\Delta F}_{c3,\Delta d}$…${\Delta F}_{c9,\Delta d}$ are related to c, but independent from $\Delta\boldsymbol{d}$. (${\Delta F}_{c,\Delta d}$=${\Delta f}_{L}(c)$)

In summary, the additional liquid resistance mainly related to target’s concentration instead of sliding directions, and the additional solid resistance mainly related to sliding direction instead of target’s concentration.

**Characterization**

The contact angles (2.0 μL), CSAs (2.0 μL) and surface tensions of the droplet were measured on an OCA20 machine (Data Physics, Germany) at ambient temperature (15-20°C). The 2.0 μL volume of RCA droplet was adopted based on our previous research. In this case, the largest difference of CSA for RCA droplets with and without 500 M ATP was achieved. To measure CSA, a substrate should be placed on the platform of the instrument and positioned exactly level, in other words 0^o^. Then, a drop of RCA droplet is placed on the surface of the substrate. Then, the OCA20 machine holding the substrate is slowly tilted and the angle at which the drop begins to slide is recorded as the CSA. The 3D profile fluorescence images were measured for Z-stack imaging on the Fluoview FV1200 confocal laser scanning microscope (Olympus, Japan), and then analyzed by FV10-ASW V4.0 Image (Olympus). The images were further dealt with Image J software to subtract background signals. The fluorescence videos of the droplets’ motion (0.3 μL) (Video S1 and S2) were monitored on inverted fluorescence microscope (Zeiss, Germany) by tilting the microscope from 0° to 9.1° with 0° sliding direction, and 30° for 90° sliding direction on the micro-grooves structural PDMS slippery surface. The droplets were dyed with 4S Green Plus (10 μM), and the PDMS slippery surface was colored with 1,1'-Dioctadecyl-3,3,3',3'-Tetramethylindodicarbocyanine, 4-Chlorobenzene sulfonate Salt (DiD) (10 μM). A 488 nm laser was chosen for the excitation of 4S Green Plus, and the emission was collected at 500-550 nm. A 635 nm laser was chosen for the excitation of DiD and the emission was collected at 640-700 nm. The videos of the droplets’ motion were recorded on two channels, FITC and Rhodamine, and presented at gray scale. The agarose electrophoresis experiment was operated with a potential of 100 V at 25°C for 70 min.

**Data Analysis**

The statistical multivariate methods, LDA, was routinely used to interpret and evaluate the droplet’s wetting properties on the anisotropic slippery surface with five different sliding directions, providing a graphical output useful to gain an insight into the clustering of the data, and calculate classification accuracy. The LDA was carried out using SYSTATH v12.02.00.

Estimation of Additional Liquid Resistance and Additional Solid Resistance

For a droplet to move on a surface tilted at an angle *α*, the tangential component of the gravitational force, mgsin*α*, acting on the droplet has to exceed the resistance, *f*. In our experiment, *α* equals to CSA, and *f* is the total resistance coming from liquid phase (liquid resistance) and solid phase (solid resistance). Therefore, for RCA droplets with various ATP concentration in different sliding directions, the total resistance (*f*) they suffered can be calculated by corresponding CSA, as shown in **Table S4**. In this experiment, when RCA droplets slide in direction of 0° with ATP concentration of 40 mM, the RCA droplet was suffered the lowest solid resistance and the lowest liquid resistance. Considering this case as a ground state, for RCA droplets slide in other directions or/and contain lower centration of ATP, they will be suffered additional resistance from solid surface or/and liquid phase. Additional solid resistances of RCA droplets with the same ATP concentration were calculated by compared the resistance between 0° and other sliding directions: 2.22 ± 0.16, 4.45 ± 0.38, 5.61 ± 0.55, 5.61 ± 0.55, 9.67 ± 0.64 μN for direction of 0°, 30°, 45°, 60°, and 90°, respective. This result demonstrates the solid resistance depends on RCA droplet’s sliding direction. Meanwhile, additional liquid resistances of RCA droplets with the different ATP concentration were calculated by compared the resistance between ATP concentration of 40 mM and other concentration: 0.02 ± 0.02, 0.27 ± 0.14, 0.46 ± 0.19, 0.84 ± 0.23, 1.21 ± 0.35, 1.74 ± 0.38, 2.42 ± 0.41, and 2.79 ± 0.63 μN for ATP concentration of 4 mM, 300 μM, 30 μM, 3 μM, 300 nM, 30 nM, 3 nM, and 300 pM, respectively (Tables S5-S7).

**Reference**

1. Scriven LE and Sternling CV. The Marangoni effects. *Nature* 1960; **187**: 186-8.

2. Gao ZF, Liu R and Wang J *et al.* Controlling droplet motion on an organogel surface by tuning the chain length of DNA and its biosensing application. *Chem* 2018; **4:** 2929-43.

3. Trantum JR, Baglia ML and Eagleton ZE *et al.* Biosensor design based on Marangoni flow in an evaporating drop. *Lab Chip* 2014; **14**: 315-24.

4. Wang JH, Huang Y and You K *et al.* Temperature-driven precise control of biological droplet's adhesion on a slippery surface. *ACS Appl Mater Interfaces* 2019; **11**: 7591-9.
